# Food Matrix as a Modulator of Spice Phytochemical Bioactivity: Evidence from In Vitro, Animal, and Human Studies

**DOI:** 10.3390/nu18183048

**Published:** 2026-09-17

**Authors:** Karolina Rak, Paweł Serek, Paulina Sławińska, Ewa Raczkowska

**Affiliations:** Department of Human Nutrition, Faculty of Biotechnology and Food Science, Wrocław University of Environmental and Life Sciences, 37 Chełmońskiego Street, 51-630 Wrocław, Poland; karolina.rak@upwr.edu.pl (K.R.); pslawinska.dietetyk@gmail.com (P.S.); ewa.raczkowska@upwr.edu.pl (E.R.)

**Keywords:** food matrix, spices, polyphenols, phytochemicals, bioavailability, functional foods

## Abstract

Spices contain numerous bioactive compounds with antioxidant, anti-inflammatory, antimicrobial, and metabolic effects. However, their biological activity depends strongly on the food matrix, which influences phytochemical stability, bioaccessibility, and physiological responses. This review evaluates the complex interactions between phytochemicals from selected spices—turmeric, ginger, cinnamon, and cloves—and various food components, including proteins, lipids, dietary fiber, and sweeteners. Drawing on evidence from in vitro models, animal studies, and human trials, the scope encompasses the effects of processing, encapsulation, and fermentation on bioactivity. The findings demonstrate that food matrices substantially modify the functionality of spice phytochemicals, rather than being merely passive carriers. Lipids, proteins, and dietary fiber improve compound stability and bioavailability, while fermentation and encapsulation further modulate their release and activity. Notably, dairy-rich systems significantly enhance the bioavailability of lipophilic compounds, such as curcuminoids. Human studies indicate that spice-containing foods may improve postprandial metabolic responses, endothelial function, oxidative status, and gut microbiota profiles. However, clinical evidence remains limited, with most human studies focusing on acute interventions rather than long-term effects. Future research must shift toward long-term clinical trials to establish chronic health benefits and explore a broader range of spices and food matrices. Key perspectives include clarifying the role of gut microbiota in metabolizing matrix-bound polyphenols and developing optimized delivery systems. Integrating molecular approaches with functional food design will be crucial for translating these interactions into effective dietary strategies.

## 1. Introduction

Spices have been used for centuries not only as flavoring and preservative agents but also as valuable components of traditional medicine. Beyond their culinary functions, they represent one of the richest natural sources of plant-derived bioactive compounds, including polyphenols, phenolic acids, flavonoids, curcuminoids, gingerols, shogaols, phenylpropanoids, and terpenoids, which exhibit a broad spectrum of biological activities [1,2]. Increasing evidence indicates that the regular consumption of culinary spices may contribute to the prevention or management of numerous chronic diseases, including obesity, type 2 diabetes mellitus, cardiovascular diseases, neurodegenerative disorders, and certain types of cancer through their antioxidant, anti-inflammatory, antimicrobial, and metabolic regulatory properties [3,4,5].

Although the biological activities of individual spice-derived phytochemicals have been extensively investigated, their efficacy in vivo depends not only on their intrinsic chemical properties but also on the environment in which they are consumed. Most spices are not ingested as isolated compounds or supplements but rather as ingredients incorporated into complex food matrices. Consequently, the physiological effects of spice phytochemicals are determined not only by their chemical composition but also by interactions with other food constituents, including proteins, lipids, carbohydrates, dietary fiber, minerals, and water. These interactions influence the stability of bioactive compounds during processing and storage, their release from the food matrix during digestion, intestinal absorption, metabolism, and ultimately their biological activity [6,7].

The concept of the food matrix has gained considerable attention in nutritional sciences over the last decade. In this review, the food matrix is defined as the physical and chemical environment formed by the food’s macronutrients, micronutrients, structural components, and processing-related constituents, which influences the stability, release, bioaccessibility, absorption, metabolism, and biological activity of phytochemicals [8,9].

Rather than considering foods as simple collections of nutrients, the food matrix describes the physical structure of foods and the complex network of interactions among their components that collectively determine digestion, nutrient bioaccessibility, absorption kinetics, and physiological responses. The term “food vehicle” refers more specifically to the food used as a carrier or delivery system for bioactive compounds, whereas “food matrix” emphasizes the compositional and structural environment in which these compounds are embedded and with which they interact. It has become increasingly evident that identical amounts of a bioactive compound may produce substantially different biological effects depending on the food vehicle in which they are delivered [6]. Matrix-related factors such as lipid content, protein composition, dietary fiber characteristics, fermentation, thermal processing, and encapsulation technologies can profoundly modify the solubility, chemical stability, gastrointestinal release, and metabolic fate of phytochemicals [7].

Among spice-derived bioactives (turmeric, ginger, cinnamon, clove) curcuminoids, gingerols, cinnamaldehyde, eugenol, and numerous phenolic compounds provide excellent examples of matrix-dependent biological activity. Curcumin, a highly lipophilic polyphenol, exhibits poor aqueous solubility and limited oral bioavailability; however, its absorption can be markedly improved by lipid-rich matrices, dairy products, or microencapsulation technologies [10,11,12]. Similarly, the biological activity of cinnamon polyphenols, gingerols, and other spice-derived phytochemicals has been shown to vary depending on food composition, processing methods, and interactions with coexisting dietary components, affecting both their bioavailability and physiological efficacy [13,14,15].

Numerous studies have demonstrated that the food matrix influences not only the bioaccessibility and pharmacokinetics of spice phytochemicals but also their biological effects, including antioxidant capacity, glycemic control, lipid metabolism, endothelial function, inflammatory responses, gut microbiota composition, and neuroprotection. Evidence from static and dynamic in vitro digestion models, animal experiments, and human intervention trials consistently indicates that food matrices can either enhance or attenuate the health-promoting properties of spices through multiple physicochemical and physiological mechanisms [10,16,17].

Despite the rapidly growing number of studies investigating spice-enriched foods, current knowledge remains fragmented, with most reviews focusing either on individual spices or on isolated phytochemicals rather than considering the food matrix as an integral determinant of biological activity. A comprehensive synthesis of available evidence addressing matrix-dependent modulation of spice bioactivity across different experimental models is therefore lacking.

The aim of this review is to critically evaluate the current evidence regarding the influence of food matrices on the stability, bioaccessibility, bioavailability, and biological effects of selected spice-derived phytochemicals. Particular emphasis is placed on curcumin, ginger-derived compounds, cinnamon phytochemicals, and eugenol-rich spices, integrating findings from in vitro studies, animal experiments, and human intervention trials. Furthermore, the review discusses the physicochemical mechanisms underlying matrix–phytochemical interactions and highlights their implications for the design of functional foods and dietary strategies intended to maximize the health benefits of culinary spices.

## 2. Literature Search Strategy

A literature search was conducted using the electronic databases PubMed, Scopus, and Web of Science to identify studies evaluating the effects of selected spices and their bioactive compounds on the nutritional quality, stability, bioaccessibility, bioavailability, and biological effects of food products. The search included articles published in English between 2010 and 2026. The following keywords and their combinations were used: spices, bioactive compounds, polyphenols, flavonoids, turmeric, cinnamon, ginger, clove, curcumin, cinnamaldehyde, gingerol, eugenol, food matrix, bioaccessibility, bioavailability, antioxidant activity, anti-inflammatory activity, antidiabetic activity, cardiovascular effects, hypolipidemic agents, functional foods, dairy products, bakery products, cereal, meat products, beverages, protein–polyphenol interactions, lipid–polyphenol interactions, polysaccharide–polyphenol interactions. Boolean operators (“AND”, “OR”) were applied to refine the search strategy. Titles and abstracts were initially screened for relevance to the scope of the review, followed by full-text assessment of potentially eligible articles.

Original research articles and relevant review papers focusing on the incorporation of spices into food matrices and the effects on the nutritional quality, stability, bioaccessibility, bioavailability, and biological effects of phytochemicals were considered eligible. Studies not related to food applications, conference abstracts, editorials, duplicate records, and publications lacking sufficient methodological information were excluded. For in vivo studies, only interventions in which spices or spice-derived compounds were administered as part of a food, meal, or non-water beverage, or immediately together with a meal, were considered relevant to the food-matrix framework; studies based solely on isolated spice supplements or compounds administered without a food matrix were excluded from the in vivo evidence synthesis. Additional relevant references were identified through manual screening of the reference lists of selected articles.

## 3. Main Classes of Spice-Derived Bioactives

**Figure 1:**
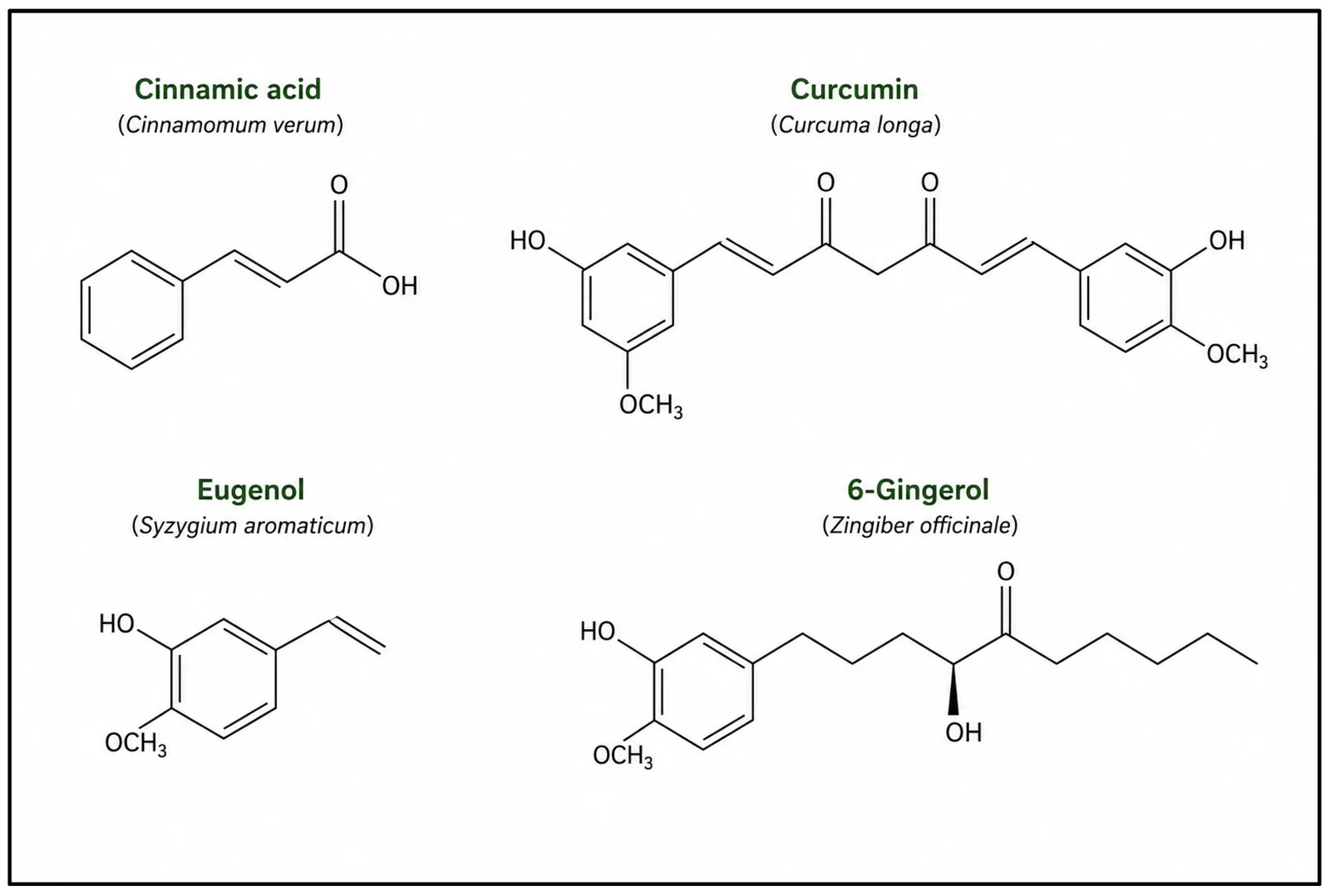
Representative chemical structures of selected spice-derived bioactive compounds.

Spices contain a diverse range of plant-derived phytochemicals that differ in their chemical structure, physicochemical properties, and biological activity. The major groups of compounds discussed in this review include phenolic acids, flavonoids and proanthocyanidins, curcuminoids, and phenylpropanoids and related compounds. Representative molecular structures of key bioactive compounds identified in commonly consumed spices are shown in Figure 1, illustrating the structural diversity of the phytochemicals discussed in the following sections.

This section provides an overview of the major classes of bioactive compounds present in cinnamon, turmeric, clove and ginger, with particular emphasis on their chemical characteristics, biological activities, and potential implications for human health (Table 1).

### 3.1. Phenolic Acids

Phenolic acids constitute an important class of polyphenolic compounds that can be extracted from a wide variety of plant materials, including culinary spices. Among spices, cloves (*Syzygium aromaticum*) and cinnamon (*Cinnamomum* spp.) are recognized as particularly rich sources of phenolic acids [21,22]. Based on their chemical structure, phenolic acids can be classified into three major groups:

- hydroxybenzoic acids (C6–C1), represented by gallic acid;
- hydroxycinnamic acids (C6–C3), including ferulic acid and caffeic acid;
- phenylacetic acids (C6–C2) [23].

Gallic acid has been identified as one of the most abundant naturally occurring polyphenols. It exhibits a broad spectrum of biological activities, including anti-inflammatory, anticancer, antifungal, and antibacterial properties [2]. Ferulic acid demonstrates diverse biological effects, including antioxidant, anti-inflammatory, anti-aging, and neuroprotective activities. Furthermore, it serves as a precursor in the biosynthesis of several biologically important compounds, including coniferyl alcohol, vanillin, and curcumin [2]. Caffeic acid is a naturally occurring antioxidant with considerable therapeutic potential. Numerous studies have demonstrated its anticancer activity, primarily through the induction of apoptosis in malignant cells and inhibition of metastatic progression [24]. In addition, caffeic acid exhibits potent antioxidant and anti-inflammatory properties [2]. It has also been shown to regulate the expression of genes involved in lipogenesis and may alleviate intestinal dysbiosis together with its associated metabolic endotoxemia and inflammatory responses [25].

### 3.2. Flavonoids and Proanthocyanidins

Flavonoids are hydroxylated polyphenolic compounds containing two or more aromatic rings connected by a heterocyclic pyran ring and at least one attached aromatic hydroxyl group. The most abundant flavonoid classes occurring in nature include anthocyanins, flavones, flavanones, flavonols, flavanonols, isoflavones, and other subclasses [2]. These compounds exhibit numerous health-promoting properties, including antioxidant, anticancer, neuroprotective, anti-inflammatory, and antimicrobial activities [26]. Proanthocyanidins, also known as condensed tannins, belong to an evolutionarily conserved group of plant secondary metabolites that are widely distributed throughout the plant kingdom and perform important biological functions. They are particularly abundant in woody plants and bark tissues. Their biological activity is associated with both their chemical concentration and molecular structure. Proanthocyanidins have been investigated for their potential role in the prevention of diabetes, cancer, and cardiovascular diseases [2]. These compounds are also present in commonly consumed spices. Cinnamon bark contains, among others, procyanidins and catechins [27]. Furthermore, studies have demonstrated that cloves exhibit strong antioxidant activity through free radical scavenging mechanisms, which is attributed to their high content of polyphenols and flavonoids [28].

### 3.3. Curcuminoids

Curcuminoids are polyphenolic pigments found in plants belonging to the genus *Curcuma*. Curcumin is the most biologically active compound among curcuminoids and is widely available commercially in the form of extracts, dietary supplements, and highly purified preparations [2]. The primary dietary source of curcumin is turmeric, a spice that has been traditionally recognized for its medicinal properties for many years [29]. Curcumin is a highly pleiotropic molecule, which is attributed to the complexity of its molecular structure and its ability to modulate multiple signaling molecules and pathways. Numerous studies have demonstrated its potential therapeutic effects in cancer, neurological disorders, metabolic diseases, autoimmune conditions, cardiovascular diseases, and inflammatory disorders affecting the lungs and liver, among others [30]. Curcumin exhibits greater stability under acidic and neutral conditions compared to alkaline environments. Its anti-inflammatory activity is associated, among other mechanisms, with the inhibition of nuclear factor kappa B (NF-κB) activation, leading to the downregulation of genes encoding pro-inflammatory cytokines, including tumor necrosis factor-alpha (TNF-α), interleukin-1β (IL-1β), and interleukin-6 (IL-6). Furthermore, the potential application of curcumin in cancer therapy is attributed to its ability to induce apoptosis in cancer cells and inhibit metastatic processes [2].

### 3.4. Phenylpropanoids and Related Compounds

Phenylpropanoids are secondary metabolites derived from glycolysis and the pentose phosphate pathways [31]. They are primarily found in plants, including culinary spices. These compounds exhibit a broad spectrum of biological activities, including anti-inflammatory, antioxidant, antiviral, anticancer, and anti-aggregatory effects [32]. Representative phenylpropanoids include eugenol, which is present in cloves and cinnamon, as well as cinnamaldehyde and cinnamic acid, which are characteristic constituents of cinnamon. These compounds contribute significantly to the antioxidant properties of the aforementioned spices [33]. Moreover, cinnamaldehyde and eugenol exhibit moderate cytotoxic and antimicrobial activities. Cinnamaldehyde also demonstrates lipoprotective effects and possesses metal ion-chelating properties [33]. Cinnamic acid has been associated with beneficial effects on carbohydrate metabolism, including the stimulation of insulin secretion, delayed carbohydrate digestion and glucose absorption, as well as improved pancreatic β-cell function [34].

Gingerols are pungent phenolic compounds found in the rhizomes of ginger (*Zingiber officinale*) [35]. In fresh ginger, gingerols, including 6-gingerol, 8-gingerol, and 10-gingerol, represent the major polyphenolic constituents. During thermal processing or prolonged storage, gingerols may undergo conversion into shogaols [36]. Both gingerols and shogaols exhibit a wide range of biological activities, including anticancer, antimicrobial, antioxidant, anti-allergic, and anti-inflammatory effects [35].

## 4. Mechanisms of Bioactivity Modulation

Food matrices consist of a complex network of biomolecules, including proteins, carbohydrates, lipids, and polyphenols, which are capable of interacting with one another. These interactions can positively or negatively influence the stability, bioaccessibility, bioavailability, and biological activity of the individual components, thereby affecting their overall physiological effects.

### 4.1. Polysaccharides—Polyphenols Interactions

Interactions between polyphenols and polysaccharides lead to the formation of complexes that alter the properties of both molecules, and are classified into non-covalent (conjugation) and covalent (complexation) categories [37]. Within the food matrix, reversible and low-energy non-covalent interactions predominate. These include hydrogen bonds [38], hydrophobic interactions [39], as well as stabilizing electrostatic forces [40], van der Waals forces [41], and π-π stacking interactions [42]. Covalent bonds form stable, irreversible linkages that significantly modify the stability and functionality of the complexes. This mechanism occurs primarily under oxidative or stress conditions, typically in damaged plant cells or during food processing [37,43].

The strength and nature of polysaccharide–polyphenol interactions depend directly on the structural characteristics of both molecules. Polyphenols with higher molecular weight and a higher degree of polymerization (e.g., procyanidins, tannins) bind to polysaccharides much more strongly than monomeric forms, offering a denser network of potential binding sites [39]. In the case of polysaccharides, linear polymer chains exhibit greater adsorption capacity for polyphenols than highly branched structures [44]. Furthermore, the strength of the interaction increases with the degree of galloylation of the phenolic compounds [39]. Environmental factors such as extreme pH values, high temperature, and the presence of specific salts can weaken noncovalent hydrogen bonds and hydrophobic interactions, promoting dissociation of the complexes [45] or—in the case of high temperatures and oxygen access—accelerating covalent cross-linking [43].

Polyphenol–polysaccharide interactions form complexes that significantly modulate their physicochemical and biological properties. This complexation is an effective strategy, improving the stability, solubility, and bioavailability of inherently unstable, hydrophobic polyphenols [39]. These complexes protect polyphenols during gastrointestinal transit, allowing controlled intestinal release [46], while carriers like cyclodextrins directly enhance aqueous solubility [47]. Consequently, they exhibit superior bioactivity, showing potential in managing diabetes [48], reducing astringency (e.g., in wine) [49], and developing active packaging that limits oxidation and microbial growth [50]. However, polysaccharide–polyphenol interactions can also have negative effects. Plant cell wall polysaccharides can physically trap polyphenols, restricting their gastrointestinal release unless processed [51,52]. In the upper gut, indigestible fibers sequester bound polyphenols, shifting absorption to the colon and reducing direct bioaccessibility [53]. Additionally, viscous soluble fibers establish a rheological barrier that impairs the diffusion of free polyphenols toward enterocytes [54]. Finally, hydrogen bonding shields active phenolic hydroxyl groups, suppressing their immediate antioxidant capacity [55].

### 4.2. Proteins—Polyphenols Interactions

Polyphenol–protein interactions form complexes altering the structural, functional, and biological properties of both molecules through non-covalent or covalent pathways. Reversible, low-energy non-covalent interactions occur under physiological conditions, involving multiple simultaneous forces. These include hydrogen bonds between polyphenol hydroxyl (-OH) groups and protein backbone/side-chain atoms (C=O, -NH, -OH); hydrophobic interactions between polyphenol aromatic rings and nonpolar residues (Leu, Ile, Phe); and electrostatic forces between charged phenolate groups and protein domains [56,57]. Complexes are stabilized by van der Waals forces and π-π stacking between aromatic rings and tryptophan, tyrosine, or phenylalanine residues [58,59]. Covalent bonds form stable, irreversible adducts in two stages. First, polyphenols oxidize into reactive quinones or semiquinones, a process driven by enzymes (e.g., polyphenol oxidase), free radicals, or alkaline conditions (pH ≥ 9.0). Second, these intermediates react with nucleophilic groups on protein side chains, targeting cysteine sulfhydryl (-SH) and lysine amino (-NH_2_) groups [57]. This proceeds via Michael addition (direct attachment of nucleophilic side chains to the quinone, forming stable C–S or C–N bonds) [60] or Schiff base formation (condensation of quinone carbonyls with lysine ε-amino groups to form an imine C=N bond) [61].

The nature and strength of protein–polyphenol interactions depend on the polyphenol structure, their relative ratio, and environmental conditions. Polyphenol structure significantly influences binding affinity; higher molecular weight molecules (such as tannins) provide more binding sites, promoting simultaneous multi-site binding and protein cross-linking [59]. Additionally, a higher number of hydroxyl groups, along with catechol or galloyl moieties, promotes covalent bonding [62], while a larger aromatic surface enhances hydrophobic and π–π stacking interactions [63]. The polyphenol-to-protein ratio also determines complex characteristics: low ratios allow polyphenols to act as multivalent ligands that cross-link multiple proteins, whereas high ratios reduce this cross-linking extent [64]. Environmental factors dictate bond reversibility. Non-covalent interactions predominate under acidic conditions (pH < 7.0), while alkaline environments (pH ≥ 9.0) favor irreversible covalent binding [65]. Heated systems accelerate polyphenol oxidation and induce protein denaturation, exposing hydrophobic cores and reactive residues to promote covalent conjugation [66]. Finally, ionic strength shifts alter electrostatic interactions by neutralizing molecular surface charges, modulating overall binding strength [61].

Protein–polyphenol complexes significantly enhance the stability, bioaccessibility, and bioavailability of polyphenols in food systems and the human body. Proteins shield unstable, free polyphenols from degradation caused by light (including UV), elevated temperatures, and storage oxidation. Covalent bonds are particularly effective, establishing a stable protective barrier known as the “structural shielding” effect [65,67]. Acting as targeted delivery platforms, protein carriers slow polyphenol release in the stomach, protecting them from gastric acidity and ensuring efficient delivery to the intestine [68,69]. Additionally, proteins increase polyphenol solubility within the micellar environment of the small intestine, directly improving overall bioavailability [70]. These complexes also exhibit synergistic biological activities. Protein complexation enhances the antioxidant and anti-inflammatory properties of polyphenols, partly by restoring endogenous enzymes such as superoxide dismutase (SOD), catalase (CAT), and glutathione peroxidase (GSH-Px) [71]. Furthermore, protein-bound polyphenols show superior anticancer [67] and hypoglycemic activities [72], supporting the management of chronic diseases. Lastly, protein–polyphenol binding can mask or modify IgE-binding epitopes, potentially reducing the allergenicity of dietary proteins found in milk or eggs [73]. However, protein–polyphenol interactions can reduce the nutritional value and quality of food. Polyphenols non-covalently inhibit digestive enzymes, limiting macronutrient absorption [74], and cross-link proteins into insoluble aggregates, reducing their digestibility [75]. Furthermore, complexation shields the active phenolic groups, suppressing antioxidant properties [75], and induces colloidal instability, leading to cloudiness in beverages [76].

### 4.3. Lipids—Polyphenols Interactions

The interactions between polyphenols and lipids depend on both polyphenol structure and membrane lipid composition, playing a vital role in determining polyphenol bioactivity. These interactions are predominantly non-covalent, involving hydrogen bonding, hydrophobic forces, van der Waals forces, electrostatic, and dipole–dipole interactions [77]. Hydrogen bonds are the primary force stabilizing polyphenol position and orientation within the bilayer; they form between polyphenol hydroxyl (-OH) groups (donors) and lipid phosphate (–PO_4_^2−^) or carbonyl (C=O) head groups (acceptors) [78]. Meanwhile, hydrophobic interactions drive polyphenol penetration into the hydrophobic core, with van der Waals forces providing structural stabilization [78]. Lastly, electrostatic and dipole–dipole forces, characteristic of flavonoids, modulate the initial adsorption of polyphenols onto the polar membrane surface [79,80].

Polyphenol–lipid interactions are governed by chemical structures and environmental conditions. Hydrophobicity (Kow) is crucial; greater lipophilicity enhances membrane adsorption, cellular uptake, and core penetration [81]. Structurally, methylation increases hydrophobicity, while glycosylation decreases it [82,83]. The hydroxylation degree balances lipophilicity and hydrogen-bonding capacity to modulate membrane affinity [84], whereas high polymerization hinders bilayer penetration due to steric constraints [85]. The lipid matrix structure dictates binding site availability and complex stability. Polyphenols prefer fluid bilayers rich in unsaturated fatty acids over highly ordered, cholesterol-enriched membranes [86]. High cholesterol reduces the partition coefficient (Kp), displacing polyphenols to the polar head-group region, which limits their antioxidant efficacy against fatty acids in the core [87]. Furthermore, polyphenol concentration and hydrophobicity dynamically alter bilayer properties [84,88]. External factors like pH, temperature, and ionic strength further modulate these interactions. Environmental pH controls polyphenol ionization and stability; at low pH, stable polyphenols penetrate deeper into hydrophobic regions, whereas neutral or alkaline pH triggers deprotonation, anchoring them to polar head-groups [89]. High temperatures accelerate oxidation and alter lipid fluidity [90], while increased salt concentrations enhance bilayer incorporation, which decreases in strongly negatively charged membranes [91].

Dietary fat enhances the bioavailability of lipophilic polyphenols by shielding them from gastric acidity [92], stimulating bile-mediated micelle formation to increase solubility and enterocyte uptake [93], and increasing epithelial permeability via lipid hydrolysis products [94]. Additionally, lipid-based formulations promote lymphatic transport, bypassing first-pass metabolism [95]. These lipid–polyphenol interactions also modulate bioactivity, stabilizing food lipids through radical scavenging and regulating lipid metabolism [96]. Furthermore, polyphenol accumulation in lipid rafts triggers intracellular signaling, while interactions with lipids and polysaccharides modulate the gut microbiota to produce highly active metabolites [9,97,98]. Conversely, these interactions can exert negative effects. In emulsions, polyphenols can act as pro-oxidants by reducing transition metals (Fe^3+^ to Fe^2+^) and undergoing autoxidation, propagating lipid peroxidation [99]. Moreover, lipophilic polyphenols can get sequestered within undigested lipid fractions, preventing micellarization and reducing bioaccessibility [93]. Finally, elevated polyphenol concentrations can perturb lipid bilayers, disrupting membrane fluidity and inducing cell leakage or death [77].

## 5. Influence of Food Matrices on the Biological Effects of Selected Spices in In Vitro Studies of Functional Foods

Research findings indicate that the food matrix is a key determinant of the nutritional efficacy and health-promoting potential of spices incorporated into foods. The physical structure of the product, its macronutrient composition, and the technological processes applied substantially influence the stability, bioaccessibility, bioavailability, and biological effects of spice-derived bioactive compounds [100,101,102,103,104]. Table 2 presents the influence of food matrices on the biological effects of selected spices in in vitro studies of functional foods.

For clarity, three related but distinct concepts are used throughout this review. Bioaccessibility refers to the fraction of a bioactive compound that is released from the food matrix during gastrointestinal digestion and becomes available for intestinal absorption. Bioavailability refers to the fraction that is absorbed and reaches the systemic circulation in an intact form or as metabolites. Bioactivity refers to the biological effects exerted by the parent compound and/or its metabolites at the cellular, tissue, or systemic level [139]. Therefore, increased bioaccessibility does not necessarily imply increased systemic bioavailability or enhanced physiological activity.

### 5.1. Cereal and Bakery Products as Carriers of Bioactive Spice Phytochemicals

Cereal and bakery products constitute suitable vehicles for delivering bioactive compounds, including dietary fiber and phenolic compounds from spices. Owing to their composition, they are rich in carbohydrates (mainly starch) and contain moderate proteins, including gluten responsible for dough structure. During baking, these formulations undergo complex physicochemical transformations (such as the Maillard reaction, caramelization, starch gelatinization, and protein denaturation) that shape sensory properties and generate both beneficial antioxidant melanoidins and undesirable contaminants like acrylamide and furan. Fortifying these products with spices can modulate these processes through interactions with the cereal matrix, influencing antioxidant capacity, quality, and safety [140,141,142,143]. Consequently, bakery formulations represent a valuable model for investigating the technological, functional, and health-promoting effects of spice enrichment.

Fortifying bakery products with either cinnamon or ginger enhances its overall nutritional profile by increasing protein, dietary fiber, and mineral content, while significantly boosting both total phenolic content (TPC) and antioxidant activity [105,106,107,119,120].

Enrichment of oil cakes with aqueous cinnamon bark extract (0.05–0.25% *w*/*v*) significantly increased TPC, total tocopherol compounds (TTC), DPPH radical scavenging activity, and ferric reducing antioxidant power (FRAP) in a dose-dependent manner [107]. In wheat flour cookies supplemented with ginger powder (2–14%), the formulation containing 12% ginger powder was optimized as the most effective blend, significantly elevating TPC, DPPH radical scavenging capacity, fiber, and mineral ash content. Sensorially, this 12% level achieved the highest overall acceptability score [121]. Similarly, fortifying wheat bread with ginger powder (2–8%) linearly elevated TPC, DPPH, and key mineral concentrations (Na, Ca, K, Fe, Zn, Cu). Addition of 2–4% ginger powder was determined as the optimal supplementation level, enhancing antioxidant and mineral value without compromising dough rheology or sensory acceptability [119]. Supplementing wheat flour cookies with ginger powder (2–14%) significantly enhances their physical, nutritional, and sensory quality, with the 12.0% ginger powder formulation optimized as yielding superior overall product characteristics. 12.0% ginger cookies exhibited marked increases in TPC, antioxidant activity, fiber, ash content, fat, protein, and total energy relative to control cookies [120].

Aromatizing oil-free pumpkin chips with 1% (*w*/*w*) powdered spices significantly increased native total phenolic content (TPC) from 7628.5 mg GAE/kg dw in controls to 8939.3 mg GAE/kg dw in ginger chips and a peak of 11,023.8 mg GAE/kg dw in cinnamon chips. This phenolic increase yielded markedly higher native antioxidant activity across ABTS, CUPRAC, DPPH, and FRAP assays, with cinnamon consistently delivering the highest values. Additionally, both spices masked raw pumpkin off-flavors, significantly improving overall consumer acceptability [135].

Interestingly, the same spice can exert highly divergent effects depending on the specific type of baked product. For instance, while incorporating 1% cinnamon into wheat bread reduced carcinogenic furan derivatives by 75% and completely eliminated 2-pentylfuran [106], adding it (0.06–0.24%) to cookies paradoxically increased acrylamide levels from 61.86 to 212.28 ng/g [108]. This undesirable increase is attributed to the high-carbohydrate matrix combined with cinnamaldehyde, which accelerates pathways leading to acrylamide formation during thermal processing [144]. In contrast, ginger in biscuits exhibited the opposite effect, suppressing acrylamide formation in a dose-dependent manner (by up to 23.7% at a 7% supplementation level) [122]. Rather than removing preformed acrylamide, ginger prevents its synthesis through a mechanism driven primarily by the phenolic hydroxyl group of 6-gingerol [122]. Specifically, ginger’s phenolic constituents (6-gingerol, 6-shogaol, and zingerone) react with methylglyoxal (MGO), a highly reactive dicarbonyl generated during glucose degradation, thereby scavenging free MGO, limiting the Maillard reaction, and indirectly reducing acrylamide formation [145].

### 5.2. Dairy Products as Carriers of Bioactive Spice Phytochemicals

Dairy products represent a suitable vehicle for delivering bioactive phytochemicals derived from spices. Due to their complex matrix rich in high-quality proteins (primarily caseins and whey proteins), milk fats, lactose, and essential minerals, dairy systems substantially improve the stability, bioaccessibility, and functional profile of incorporated spice-derived compounds.

Fortification of various dairy and dairy-like matrices with spice bioactives enhances total phenolic content (TPC) and radical scavenging activity in a dose-dependent manner. In UHT milk, 0.9–2.8% turmeric powder increases DPPH radical scavenging from 49.96% to 81.76–90.51% [128]. Fermented milk containing 20% *w*/*v* aqueous extracts of cinnamon bark (CB) or cinnamon twigs (CT) showed marked increases in TPC, DPPH, and FRAP, higher for CB [109]. Fortifying stirred yogurt with 0.8% *w*/*v* waste cinnamon leaf aqueous extract (CLE) boosts pre-digestion TPC and TFC 4.5–5 fold (TPC reaching 0.27–0.29 mg GAE/mL vs. 0.06 in control) and DPPH 2.7–3.2 fold [110]. Similarly, 0.1–0.3 g/mL aqueous cinnamon extract enriches yogurt TPC, TFC, and DPPH [112], while 0.5–1.5% cinnamon extract raises set-yogurt TPC from 3.68 to 21.80–42.66 mg GAE/100 g and DPPH up to 67.29% [114]. Supplementing fermented milk yogurt with 0.5–2.5% ginger powder similarly leads to notable increases in total phenolic content (TPC), DPPH radical scavenging activity, and overall protein and fat content [123]. The fortification of reduced-fat ice cream with processed ginger (0.5–20% in various forms, including juice, paste, powder, and candy) leads to a dose-dependent increase in total phenolic content (TPC) and DPPH radical scavenging capacity, regardless of the ginger preparation used [124]. Furthermore, a fermented dairy product formulated with 50% plantain (*Musa paradisiaca*) syrup and 0.1–0.5% turmeric powder exhibited a dose-dependent increase in TPC, enhanced water retention capacity, reduced lipid oxidation (lower TBARS values), and elevated calcium levels reaching 148.78 mg/100 g at 0.5% fortification [131].

The specific macronutrient composition of the dairy carrier strongly dictates its functional delivery efficiency. Comparative trials revealed that ultrafiltered (UF) Kariesh cheese fortified with 10% *w*/*w* aqueous turmeric extract (applied at 2% in cheese vs. 1% in skimmed milk yogurt) yielded higher TPC (14.74 mg/g vs. 4.32 mg/g), total flavonoid content (TFC: 4.83 mg/g vs. 1.39 mg/g), and radical scavenging activity (75.36% vs. 26.72%) than the corresponding yogurt, owing to the higher protein, dry matter, and fat content of the cheese matrix [130].

Interestingly, fortifying cow’s milk yogurt with 1% ethanol cinnamon extract provides an antidiabetic effect by 97.79% alpha-glucosidase inhibition (with the raw extract demonstrating an IC_50_ of 35.37 ppm) [113].

Spice bioactives within dairy matrices exhibit potent antimicrobial activity against foodborne pathogens [146,147,148]; however, their influence on lactic acid fermentation remains highly dependent on both dose and matrix form.

The addition of 0.5–1.0% turmeric powder significantly reduced yogurt starter bacteria (*L. bulgaricus* and *S. thermophilus*) viability compared to plain yogurt (3.4–3.5% vs. 1.9% loss after 15 days of storage) [13]. Similarly, incorporation of 0.5% powder caused an initial ~12% decrease in lactobacilli, whereas a 0.75% concentration resulted in the lowest lactobacilli count despite exhibiting the most favorable overall microbiological profile after 12 days [149]. Turmeric’s turmerones and curcumin disrupt bacterial cell membranes and metabolism, exerting a dose-dependent inhibitory effect on sensitive Gram-positive starter cultures [13,101]. Nevertheless, despite population reductions, beneficial yogurt microbiota consistently remain within required regulatory limits (≥6 log CFU/g) [150]. Conversely, a diluted aqueous turmeric extract (1% in yogurt, 2% in Kariesh cheese) significantly stimulated starter culture growth (*S. thermophilus*, *L. bulgaricus*) and lactic acid production [130]. This positive effect likely reflects the substantially lower effective dose compared to concentrated powder.

A distinctly different effect was observed in cinnamon-enriched products. Adding aqueous cinnamon extract (0.1 g/mL) significantly stimulated *Bifidobacterium bifidum* in cow’s and camel’s milk yogurts, elevating initial populations to 6.6 × 10^9^ CFU/mL and 25.55 × 10^9^ CFU/mL, respectively (vs. 1.9 × 10^9^ CFU/mL in control). Both fortified yogurts maintained superior probiotic viability above regulatory standards (≥6 log CFU/g) throughout two weeks of refrigerated storage [151]. Infusing aqueous cinnamon extract into yogurt provides essential minerals and nutrients that enhance starter culture metabolic activity, accelerating sugar conversion to lactic acid. Consequently, rapid acidification occurs, with titratable acidity rising to 1.54% compared to 0.86% in plain control yogurt [112].

In fermented milk supplemented with 20% (*w*/*v*) aqueous cinnamon bark or twig extracts, lactic acid bacteria exhibited (*L. bulgaricus*, *S. thermophilus*, and *L. plantarum*) intensified sugar consumption, with cinnamon bark extract increasing titratable acidity to 0.95% (vs. 0.81% in control), confirming a stimulatory effect on fermentation [109].

Cinnamon fortification (0.3–2.5%) significantly altered microbial dynamics in propolis-containing yogurt by counteracting propolis-induced inhibition of *B. animalis* subsp. *lactis* and *L. bulgaricus*, while simultaneously exerting a strong inhibitory effect on *Streptococcus thermophilus* [152].

### 5.3. Meat Products as Carriers of Bioactive Spice Phytochemicals

Meat products represent a suitable matrix for the delivery of spice-derived bioactive compounds due to their high content of proteins, lipids, and water, which strongly influence oxidative stability and microbial growth during processing and storage. Consequently, meat products provide a valuable model for investigating the effects of spice fortification on product quality, shelf life, and health-promoting properties, provided that appropriate addition levels are maintained. In meat matrices, spices interact with meat lipids and proteins, acting primarily as natural stabilizers against oxidative deterioration.

In frozen chicken nuggets, the direct addition of 600 ppm clove essential oil (CEO) reduced thiobarbituric acid reactive substances (TBARS) by 18.53% (to 1.06 mg MDA/kg) and peroxide value (PV) by 22.56% over 90 days of storage, whereas a 0.1% CEO formulation in raw buffalo patties lowered TBARS by 73% compared to controls [153,154]. Similarly, incorporating 1–2% ginger powder significantly enhances the total antioxidant capacity (ABTS, DPPH, FRAP) of both raw and cooked meats, suppressing lipid oxidation (TBARS) during refrigerated storage at 4 °C and subsequent thermal processing [126,127]. Crucially, these spice phytochemicals retain their antioxidant potential and lipid peroxidation inhibitory efficacy even after exposure to thermal processing (across both high and low temperatures), ensuring sustained protection in ready-to-eat products.

Furthermore, ginger supplementation enriches the matrix with polyunsaturated fatty acids (PUFA), increases the ω3/ω6 ratio, and reduces saturated fatty acids (SFA), thereby improving the atherogenicity (AI), thrombogenicity (TI), and h/H health indices in pork and rabbit products. Additionally, its antimicrobial activity delays the growth of total aerobic bacteria and *Pseudomonas* spp., extending shelf-life without compromising sensory acceptance [126,127].

At high dosage, however, spices can impair meat sensory acceptance and technological characteristics [155,156,157].

### 5.4. Beverages and Plant-Based Products as Carriers of Bioactive Spice Phytochemicals

Beverages function as effective food matrices and liquid carrier systems for spice-derived phytochemicals, such as cinnamaldehyde, eugenol, gingerols, shogaols, and curcuminoids, delivering health-promoting bioactive properties while modifying beverage stability and sensory attributes. Furthermore, added sweeteners significantly modulate these functional and biological characteristics.

Functional (water-based) beverages fortified with spices exhibit a very high total phenolic potential (TPC) and high antioxidant capacity. In soursop (*Annona muricata*) leaf tea, adding cinnamon powder (10–30%) increases DPPH radical scavenging activity from an initial 10.05% to 29.19% due to the introduction of cinnamaldehyde and flavonoids [118]. Similarly, in coffee-ginger beverages, the initial level of TPC (2.69 mg GAE/mL) and DPPH radical scavenging (0.43 mmol TE/mL) is highest when using non-decaffeinated Robusta coffee (*Coffea canephora*) extract blended with ginger, whereas decaffeination treatment reduces these parameters due to the breakdown of chlorogenic acid [125]. The traditional aqueous cinnamon beverages (2% cinnamon bark) exhibit a high total phenolic potential (~43.8 mg CE/g) and a strong baseline ABTS antioxidant capacity (~53.7 mg CE/g). Significantly, adding sweetening agents like sucrose (2.5% *w*/*v*) or honey (5% *w*/*v*) does not negatively alter these parameters [117]. In the traditional aqueous beverage Wedang Uwuh (ginger 65.1%, sappan wood 19.6%, clove 7.3%, nutmeg 6.1%, clove 4.9% *w*/*v*), extending the boiling duration to 15 min achieves the highest initial TPC (65.48 mg GAE/serving) and the highest antioxidant capacity measured by DPPH (96.17 mg TE/serving) and FRAP (62.25 mg TE/serving) assays, which results from thermal cell wall disruption and the subsequent release of compounds into solution [138]. Adding 1 g/L cinnamon to Roselle (*Hibiscus sabdariffa*) beverages increases initial phenolics and FRAP capacity while stabilizing anthocyanins and color. At 40 °C, cinnamon extends anthocyanin half-life (t_1/2_) from 7 days (control) to 13 days alone, and 21 days with 80 g/L sucrose (vs. 12 days with stevia) via co-pigmentation and acylation. Conversely, 1 g/L ginger offers no protection, yielding shorter half-lives (5–6 days), unmitigated antioxidant loss, and storage browning [137].

Evaluating turmeric addition (0.4–2.0%) in SCOBY-fermented black tea kombucha identified 0.8% as the optimal formulation. This concentration achieved the highest TPC (147.45 µg GAE/mL), the highest microbial load (2.5 × 10^7^ CFU/mL) and *E. coli* inhibition (3.13 mm) compared to standard black tea kombucha. Exceeding 0.8% turmeric suppressed fermentation process, microbial load (e.g., 1.1 × 10^7^ CFU/mL for 2.0%), and antibacterial efficacy (e.g., 1.25 mm of *E. coli* inhibition zone for 2.0%) due to excess antimicrobial curcuminoids [101]. Overall, moderate turmeric fortification (0.8%) maximizes the bioactivity and antibacterial efficacy of kombucha, whereas excessive addition impairs overall beverage quality.

In plant-based solid model matrices (e.g., vegan white chocolate), incorporating 2% encapsulated cinnamon extract (ECE) increases TPC to 194.63 µg GAE/g and TFC to 37.49 µg QE/g, while lowering the IC_50_ value in the DPPH assay to 37.29 mg/mL, confirming substantially enhanced free radical neutralizing capacity [116]. Enriching tiger nut yogurt with cinnamon extract (optimally at a 98:2 ratio) lowers pH, significantly boosts essential mineral levels (P, K, Na, Fe, and Zn), and enhances overall sensory acceptability by masking tannin bitterness [115].

### 5.5. Post-Digestion Effects of Phytochemicals and Matrix Macronutrients Across Diverse Food Matrices

The choice of in vitro digestion model critically determines the observed stability, release, and bioactivity of dietary phytochemicals. Static models (e.g., the standardized INFOGEST protocol) rely on fixed enzyme-to-substrate ratios, static pH endpoints, and single-step biochemical additions [13,100,103,104,109,110,117,138]. While cost-effective and high-throughput for preliminary screening, static systems fail to capture physical food matrix transformations. Conversely, dynamic gastrointestinal models—such as DIVGIS and HGS—replicate peristaltic contractions, continuous gastric emptying, dynamic pH curves, and progressive enzyme/bile secretions [111,129,158]. The physiological relevance of dynamic modeling is strikingly illustrated in carbohydrate–phytochemical interactions: cinnamon’s inhibitory effect on starch hydrolysis in rice pudding was exclusively detectable under dynamic conditions, where gradual acidification and continuous emptying preserved active cinnamaldehyde and polyphenols to interact with *α*-amylase [159]. Furthermore, dynamic models are essential for tracking physical structural changes—such as protein coagulation, curd formation, and lipid micellization—that directly regulate digesta outflow and the ultimate bioaccessibility of lipophilic bioactives [111,129,158].

Despite these advantages, static models remain dominant due to their operational simplicity and ease of cross-laboratory standardization. However, substantial variations in digestive fluid composition, pH, and enzyme activities across studies present major analytical challenges [100,104,117]. These fluctuations alter the chemical and physical structures of both phytochemicals and matrix macronutrients, directly dictating compound stability and release, which can lead to divergent interpretations of actual bioavailability.

#### 5.5.1. Dairy Matrices

Gastrointestinal digestion exerts profound reciprocal effects on the structural breakdown, hydrolysis kinetics, and overall digestibility of food matrix macronutrients. In dairy matrices, the rate of macronutrient breakdown and the gastrointestinal fate of encapsulated phytochemicals are dictated by protein network architecture and thermal pre-treatments. In dairy systems, the structural architecture of the protein network formed during gastric transit dictates the rate of macronutrient hydrolysis, lipolysis, and the post-digestive bioaccessibility of incorporated phytochemicals. Recombined skim milks enriched with a curcumin nanoemulsion show that high-heat treatment (MHH) forms a soft, open, and fragmented gastric curd that undergoes rapid digestion and gastric emptying, resulting in higher intestinal lipolysis and curcumin bioaccessibility exceeding 70% compared to low-heat (MLH) milks that form dense, cohesive clots [129].

In stirred yogurt fortified with curcumin-loaded solid lipid nanoparticles (SLN), dynamic intestinal digestion results in a lower trichloroacetic acid (TCA) solubility index (21.8–31.4% or ~22%) compared to plain control yogurt (38.2–56.6% or ~39%). In fermented dairy systems, adding curcumin-loaded solid lipid nanoparticles (SLN) to stirred yogurt reduces the duodenal and final trichloroacetic acid (TCA) solubility index from 38.2 to 56.6% (in plain yogurt) to 21.8–31.4% [111]. This reduction in protein digestibility stems from released curcumin and SLNs forming hydrophobic, ionic, and electrostatic complexes with caseins and digestive proteases (pepsin, trypsin, α-chymotrypsin), sterically hindering enzymatic cleavage [160]. Despite this slowed proteolysis, post-digestive curcumin bioaccessibility (~29.8%) and stability (~41.8%) remain fully preserved. However, digested jejunum and ileum filtrates of curcumin–SLN yogurt display cytotoxicity toward Caco-2 cells (<20% cell viability) [111], attributed to curcumin–protein aggregates, protein corona formation around nanoparticles, or amphiphilic digestion products [161,162].

In fermented milk enriched with cinnamon bark (CB) and twig (CT) extracts, intestinal digestion induces a marked decline in free phenolics and radical scavenging capacity across all formulations. Nevertheless, unencapsulated CB fermented milk retains the highest post-digestive DPPH radical scavenging activity (28.57%), while maltodextrin encapsulation successfully protects total flavonoid content (0.23 mg QE/mL) after intestinal transit [109]. In stirred yogurt fortified with waste cinnamon leaf extract (CLE), unencapsulated CLE achieves the highest post-intestinal anti-inflammatory activity, reaching 89.71% albumin denaturation inhibition (ADI) compared to 78.15% in plain control yogurt [110], driven by the synergistic action of released free phenolics and milk-derived bioactive peptides generated during gastrointestinal digestion [163]. Conversely, agar encapsulation of CLE in yogurt protects antioxidants against digestive breakdown, retaining the highest post-intestinal DPPH activity (25.31%) and ferric reducing power (63.0 µg Fe^2+^/mL), while technologically reducing syneresis to 64.21% compared to 81.84% in control yogurt without altering rotational viscosity [110]. For turmeric-enriched yogurt (1.0% turmeric powder), exposure to simulated gastric pH (3.0) and 0.5% bile salts leads to post-incubation stability losses: a 25.6% drop in DPPH radical scavenging capacity, an 11.1% loss in total phenolic content under acidic conditions, and a 12.5% loss under bile salt incubation [13].

When bovine milk (25% *v*/*v*) is incorporated into traditional Egyptian cinnamon beverages, gastro-pancreatic proteolysis hydrolyzes caseins into small peptides and free amino acids, re-releasing bound phenolics, kaempferol, and syringic acid into the micellar phase to achieve a final post-pancreatic polyphenol bioaccessibility of 80.8% (comparable to 79.8% in milk-free drinks) and a cinnamaldehyde bioaccessibility of 64% [117]. While sweeteners (sucrose and acacia honey) enhance polyphenol bioaccessibility in water (>90%) by blocking tannin–pepsin aggregation, milk addition neutralizes this sugar-induced enhancement (80.8% with milk + sucrose; 79.1% with milk + honey) due to the higher binding affinity of caseins for proanthocyanidins [164]. Concurrently, milk protein cleavage during digestion generates radical-scavenging peptides, elevating post-pancreatic ABTS radical scavenging capacity to 94.0 mg catechin/g compared to 49.9 mg/g in milk-free beverages [117].

The gastrointestinal digestion of spice-fortified dairy matrices induces structural protein breakdown, generating radical-scavenging peptides and re-releasing bound phenolics that preserve overall bioaccessibility and antioxidant capacity. However, accurate evaluation of these enriched bioactives presents notable analytical challenges due to protein–polyphenol masking and assay interference caused by milk protein breakdown products [75,102,117].

#### 5.5.2. Cereal Matrices

Physical matrix density, lipid content, and dietary fiber composition in cereal systems strongly dictate post-digestive bioaccessibility, starch digestibility, and glycemic response. Following static in vitro digestion, curcuminoid bioaccessibility is significantly higher in solid biscuits (38.8–58.8%) than in semi-fluid custards (20.9–38.8%) [104]. Higher fat and sugar levels in biscuits promote the formation of mixed bile salt–phospholipid micelles during digestion, facilitating lipophilic curcuminoid solubilization [165,166]. Specific fiber fractions further modulate this process: soluble fibers (inulin, maize dextrin) and hemicellulose-rich fibers (from yellow pea, apple, or carrot) enhance curcuminoid bioaccessibility in biscuits by stabilizing oil-in-water emulsions formed during digestion. Conversely, insoluble microcrystalline cellulose severely restricts post-digestive bioaccessibility (24.0–27.1% in custards and 39.8–42.3% in biscuits) by physically entrapping phenolics or impeding micellar transfer [104].

In baked muffins partially substituted with 2.5%, 5%, and 10% turmeric flour, released polyphenols competitively inhibit digestive disaccharidases, decreasing the post-digestion IC_50_ for *α*-amylase from 780.15 µg/mL (control) to 416.50 µg/mL (10% turmeric) and for and *α*-glucosidase from 502.65 µg/mL to 401.08 µg/mL [103]. Consequently, in vitro starch digestibility (IVSD) drops from 55.60% in control muffins to 49.07% in 10% turmeric muffins, accompanied by a reduction in estimated glycemic index (eGI) from 51.68 to 46.30 and enhanced oxidative stability, as indicated by decreased malondialdehyde (MDA) accumulation over a 14-day storage period [103].

Fortification of oil-free, hot-air-dried pumpkin chips with ginger (1% *w*/*w*) or cinnamon (1% *w*/*w*) markedly increased post-digestive phenolic bioaccessibility and antioxidant capacity across multiple assays (ABTS, CUPRAC, DPPH, FRAP). Among the spices, cinnamon fortification consistently exerted a stronger enhancing effect than ginger [135].

#### 5.5.3. Aqueous Matrices and Sweetened Beverages

In plain water-based Egyptian cinnamon beverages, post-pancreatic polyphenol bioaccessibility is limited to 79.8% due to gastric tannin–pepsin precipitation [117]. Adding sweeteners such as sucrose (1.87 g/100 mL) or acacia honey (3.75 g/100 mL) increases bioaccessibility to 92.0% and 90.6%, respectively. Sugar hydroxyl groups engage in competitive hydrogen bonding with phenolic hydroxyls, sterically preventing tannin–pepsin aggregation [167]. Sucrose is more effective than honey’s main components (glucose and fructose) at preventing these insoluble complexes [168]. However, adding bovine milk neutralizes this enhancement, returning bioaccessibility to baseline levels (~79.1–81.8%) [117] due to strong casein–proanthocyanidin binding affinity [164].

In purely aqueous multi-spice beverage such as Indonesian Wedang Uwuh (containing ginger, sappan wood, clove, nutmeg, and cinnamon leaves), static in vitro gastrointestinal digestion causes a severe, irreversible drop in TPC, FRAP, DPPH, and ABTS radical scavenging capacity across all boiling treatments (5–15 min), demonstrating the high susceptibility of aqueous spice phenolics to gastrointestinal degradation [138].

#### 5.5.4. Whole Diet Matrix

In a static in vitro digestion model evaluating ginger rhizome (*Zingiber officinale*) extract co-digested with complete daily dietary rations, macronutrient density was shown to protect active gingerols and gingerdiones during gastrointestinal transit [100]. Although digestion reduced free polyphenols by 55–99%, the bioaccessibility and post-digestive bioavailability of key metabolites were highest under a high-fiber diet (50.3 g fiber, rich in proteins, fats, and vitamins C and E). Measured via dialysis membrane diffusion, bioavailability under the high-fiber diet compared to a basic diet reached 33.29% vs. 21.32% for 6-gingerol (compared to 35.46% in a water control), 21.42% vs. 5.30% for 8-gingerdione, 6.73% vs. 2.0% for 8-shogaol, and 21.0% vs. 0.98% for 10-gingerdione [100]. While lower protein and fat contents led to a progressive decline in bioavailability, the high-fiber, nutrient-rich matrix served as an antioxidant shield and lipophilic carrier, protecting phytochemicals against oxidative degradation and cleavage. Ultimately, these findings demonstrate that a balanced food matrix serves as an essential protective vehicle for spice-derived bioactives during gastrointestinal transit [100,169,170,171].

## 6. Biological Consequences of Matrix–Phytochemical Interactions

### 6.1. In Vivo Animal Studies

Following the evidence obtained from in vitro studies, animal experiments provide a more integrated model for evaluating the influence of food matrices on the biological activity of spice-derived phytochemicals under physiological conditions. Unlike in vitro systems, animal models enable the simultaneous assessment of gastrointestinal digestion, intestinal absorption, tissue distribution, metabolism, interactions with the gut microbiota, and systemic biological responses. However, findings from animal studies should not be directly extrapolated to humans because of interspecies differences in gastrointestinal physiology, metabolism, microbiota, and dose exposure. Consequently, they offer valuable insight into the mechanisms through which food matrices modulate the in vivo efficacy of spice-derived bioactive compounds.

Consistent with the findings from in vitro studies, evidence from animal models indicates that the biological activity of spice-derived phytochemicals is determined not only by their intrinsic chemical properties or administered dose but also by the characteristics of the accompanying food matrix. Dairy products, plant protein matrices, lipid-rich diets, and complex feed formulations have all been shown to modify the bioavailability, tissue distribution, metabolic fate, and physiological effects of these compounds. The available evidence is summarized in Table 3.

#### 6.1.1. Dairy and Protein-Rich Matrices Enhance the Biological Efficacy of Spice Phytochemicals

Among the investigated food matrices, dairy products appear to be particularly effective carriers of lipophilic spice-derived phytochemicals. The protein–lipid matrix of yogurt improved the physiological activity of curcumin in streptozotocin-induced diabetic rats, resulting in significant reductions in blood glucose, triacylglycerol concentrations, and liver enzyme activities, together with partial restoration of body weight compared to untreated diabetic animals [172]. These observations are consistent with previous in vitro findings indicating that milk proteins and lipids stabilize curcuminoids during gastrointestinal digestion and improve their dispersion.

A beneficial role of dairy matrices has also been demonstrated for cinnamon-derived compounds. Drinkable yogurt containing alginate-microencapsulated cinnamon flavonoids improved glucose homeostasis and lipid metabolism in rabbits with metabolic syndrome, reducing serum glucose, total cholesterol, LDL cholesterol, and abdominal circumference [178]. Likewise, cinnamon extract incorporated into camel milk yogurt significantly reduced blood glucose, triacylglycerol, and total cholesterol concentrations in diabetic rats [179]. These findings suggest that dairy proteins, milk fat, and microencapsulation technologies may act synergistically to protect cinnamon polyphenols during digestion and enhance their biological activity.

Plant protein matrices also modify spice bioactivity. Administration of cinnamon extract incorporated into defatted soy flour rapidly decreased fasting blood glucose in mice fed a very-high-fat diet [177]. Soy proteins probably contributed to the stabilization and gradual release of cinnamon polyphenols, although the precise molecular mechanisms remain to be elucidated.

#### 6.1.2. Diet Composition Determines Metabolic Responses to Spice-Derived Phytochemicals

The metabolic effects of spice-derived phytochemicals are strongly influenced by the nutritional composition of the background diet. Most studies demonstrating beneficial metabolic outcomes employed high-fat or high-fat/high-sucrose diets, which mimic obesity and metabolic syndrome.

Curcumin degradation products administered to mice consuming a high-fat diet markedly attenuated body weight gain, hepatic steatosis, and gut dysbiosis while simultaneously increasing short-chain fatty acid production [173]. Similarly, supplementation of high-fat/high-fructose diets with 6-gingerol reduced hepatic steatosis by suppressing endoplasmic reticulum stress and NF-κB activation [173], whereas the stable analog aza-6-gingerol inhibited adipogenesis, reduced fat accumulation, and improved insulin sensitivity in mice fed a high-fat diet [176].

Comparable observations were reported for cinnamon-derived compounds. Dietary cinnamaldehyde promoted browning of white adipose tissue, reduced adiposity, and improved insulin sensitivity in obese mice [180], while supplementation of high-fat/high-sucrose diets with cinnamaldehyde dose-dependently decreased visceral fat accumulation and body mass [182]. Likewise, dietary eugenol incorporated into a high-fat diet reduced body weight gain, fasting glucose concentrations, visceral adiposity, and favorably modulated gut microbiota composition [183].

Collectively, these studies indicate that high-energy diets not only induce metabolic disturbances but also provide a physiologically relevant matrix for evaluating the metabolic efficacy of spice-derived phytochemicals. Under these conditions, spices consistently attenuate obesity-associated inflammation, dyslipidemia, insulin resistance, and hepatic steatosis.

#### 6.1.3. Matrix Composition Influences Tissue-Specific Biological Responses

Besides metabolic regulation, the food matrix also modifies tissue-specific biological responses to spice-derived phytochemicals.

Supplementation of a corn–soybean diet with curcumin improved intestinal morphology, strengthened epithelial barrier integrity, and enhanced systemic antioxidant defense in weaned piglets, as reflected by increased serum superoxide dismutase activity [17]. In contrast, curcuminoids administered as an aqueous suspension containing turmeric essential oil exerted pronounced neuroprotective effects in mice exposed to aluminum-induced neurotoxicity, improving memory performance and protecting hippocampal neurons [174].

The food matrix also influenced tissue protection in other disease models. Cinnamon incorporated into oatmeal cookies promoted regeneration of hepatic and renal tissues in rats with carbon tetrachloride-induced toxicity [181], whereas dietary supplementation with clove extract protected the heart, liver, and ocular tissues against diabetic damage [184].

These findings suggest that the biological consequences of matrix–phytochemical interactions extend beyond metabolic regulation and include modulation of intestinal integrity, antioxidant defenses, neuroprotection, and tissue regeneration. Importantly, these tissue-specific effects cannot be attributed solely to changes in systemic bioavailability, as food matrices may also influence phytochemical metabolism, metabolite formation, tissue-specific signaling, and interactions with the gut microbiota.

#### 6.1.4. Interactions Between Spice Phytochemicals Depend on the Delivery Matrix

Animal studies also demonstrate that combining different spice-derived bioactive compounds does not invariably produce additive health benefits. In diabetic rats receiving curcumin incorporated into natural yogurt, co-administration of a high dose of piperine unexpectedly abolished many of the antidiabetic and antioxidant effects of curcumin [186]. This observation contrasts with the widely accepted view that piperine universally enhances curcumin bioavailability and suggests that interactions between phytochemicals may depend on the food matrix, dose ratio, and disease model.

Conversely, when curcumin and piperine were administered as a carboxymethy–cellulose suspension in an acute pain model, a marked synergistic analgesic effect was observed [188]. These apparently contradictory findings emphasize that the biological outcome of phytochemical combinations depends not only on the compounds themselves but also on the physicochemical properties of the delivery system and the physiological context. These findings also illustrate that enhanced bioavailability of an individual phytochemical does not necessarily translate into greater biological efficacy, particularly when interactions between co-administered compounds modify downstream physiological responses.

Animal studies consistently demonstrate that food matrices are integral determinants of the biological activity of spice-derived phytochemicals. Dairy matrices, plant protein systems, lipid-rich diets, and complex feed formulations modify not only the bioavailability of these compounds but also their metabolic, antioxidant, anti-inflammatory, neuroprotective, and tissue-protective effects. Importantly, several observations—including the contrasting effects of curcumin–piperine combinations—suggest that matrix–phytochemical interactions are highly context-dependent and cannot be explained solely by changes in intestinal absorption. These findings provide an important mechanistic bridge between the controlled observations obtained in vitro and the more heterogeneous responses observed in human intervention studies.

### 6.2. In Vivo Human Studies

Regarding spices and their bioactive components administered in food matrices, the majority of available data originates from in vitro studies and animal models. However, human intervention trials investigating spices administered within food matrices represent a relatively recent and still underexplored area of research. Most published studies have focused on acute responses to spice-containing foods, beverages, or meals, whereas evidence on the effects of regular, long-term consumption remains scarce.

Existing studies vary in terms of the spice investigated, the type of food matrix, the study design, and the evaluated endpoints. Most published clinical trials are randomized crossover studies conducted in healthy adults, while individuals with overweight, obesity, or type 2 diabetes have been investigated less frequently. Turmeric has been studied most extensively, particularly in relation to curcuminoid bioavailability, whereas substantially fewer studies have evaluated ginger or cinnamon as individual spices, and no studies evaluating cloves alone were identified. Culinary spice blends have received comparatively greater attention and may better reflect habitual dietary consumption.

The most commonly evaluated endpoints included systemic bioavailability of spice-derived compounds and physiological bioactivity, assessed through postprandial glycemic and lipid responses, appetite regulation, endothelial function, oxidative stress, inflammatory response, and gut microbiota composition. The findings are presented in the following subsections according to the main physiological effects evaluated, while Table 4 summarizes the characteristics of the food matrices, study designs, and principal findings of the included human intervention studies.

#### 6.2.1. Food Matrix and Bioavailability

Among all spices, the impact of the food matrix on systemic bioavailability has been most extensively studied for turmeric and curcuminoids. This may stem from the very low bioavailability of curcuminoids when administered in an isolated form and the potential to modulate their absorption through the composition of the food matrix and processing methods [11,12].

Clinical studies indicate that both matrix type and the presence of other food constituents can significantly affect curcuminoid absorption. In a study utilizing curcumin-enriched bread, microencapsulation increased and prolonged curcumin exposure compared to the non-microencapsulated form; however, the concurrent incorporation of piperine, quercetin, and genistein did not further enhance bioavailability relative to microencapsulated curcumin alone [10].

The importance of the natural spice matrix was also confirmed in a study comparing fresh turmeric, powdered turmeric, and isolated curcumin administered with an identical meal. The highest plasma curcumin concentrations were observed following the consumption of powdered turmeric, whereas the lowest levels were recorded after the administration of the isolated compound, suggesting that co-occurring natural constituents within the spice matrix may enhance curcuminoid bioavailability [11].

The impact of various food matrices was also evaluated in a recent randomized study comparing five food products delivering an identical dose of curcuminoids. Bioavailability was shown to be significantly dependent on the product type, with the highest AUC and C_max values achieved after consuming an oat beverage. However, not all matrices produced a significant enhancement relative to the capsule reference, as the ready-to-drink nectar and pectin gummy formulations were bioequivalent to the capsule formulation. Concurrently, the metabolite profile remained similar regardless of the matrix used, indicating that under the investigated conditions, the food matrix primarily influenced the extent of absorption rather than altering the curcuminoid metabolic profile [12].

Collectively, available research indicates that an appropriately selected food matrix can substantially enhance the bioavailability of curcuminoids. At the same time, the magnitude of this effect appears to depend on the specific matrix composition and formulation, and additional bioactive constituents do not necessarily confer further enhancement. Nevertheless, clinical trials addressing the matrix effect remain restricted almost exclusively to turmeric interventions, highlighting the need to expand research to other spices and their bioactive compounds.

#### 6.2.2. Postprandial Metabolic Responses

The impact of spices administered in food matrices on postprandial metabolic responses represents one of the most extensively investigated areas of clinical research. Available studies have focused primarily on assessing glycemia, insulinemia, lipemia, and appetite-regulating hormones, with observed effects depending on spice type, dosage, the food matrix employed, and the target population.

Studies utilizing individual spices have yielded varied metabolic outcomes. In a study where a turmeric beverage was administered prior to a standardized meal, no significant effect on subjective appetite sensations was observed. Instead, differences in satiety were driven by the fat content of the meal rather than turmeric consumption [189]. Divergent results were obtained for a breakfast enriched with turmeric and black pepper—the addition of black pepper, both alone and combined with turmeric, reduced postprandial glycemia as well as reported hunger and prospective consumption, whereas turmeric alone elicited no comparable changes [192]. In the only identified study evaluating ginger as a distinct intervention condition, no significant impact on postprandial energy expenditure, appetite, or energy balance was demonstrated [190].

Favorable effects have also been observed when incorporating spices directly into food products. Both yogurts containing cinnamon or turmeric oleoresin reduced peak glucose concentration and the area under the glycemic curve compared to standard yogurt [15]. Similarly, spice beverages consumed before breakfast demonstrated composition-dependent actions—beverages containing turmeric and cinnamon attenuated early glycemic responses, whereas the turmeric beverage additionally increased postprandial peptide YY (PYY) concentrations and reduced the desire to eat [191]. Furthermore, consumption of TopiGluk bread, containing a multicomponent herbal and spice blend, resulted in lower postprandial glucose and insulin concentrations in participants with type 2 diabetes, whereas no corresponding differences were observed in healthy participants [193].

Several studies have evaluated culinary spice blends, including the Polyspice program using a curry prepared from seven spices and four vegetables. The intervention demonstrated a dose-dependent reduction in postprandial glycemic response [199], a dose-dependent increase in the total postprandial GLP-1 AUC [197], and a reduction in subjective hunger and desire to eat without significant changes in ghrelin levels [198].

Other studies evaluated standardized spice blends incorporated into high-fat, high-carbohydrate meals. Initial studies demonstrated reductions in postprandial insulin and triglyceride concentrations, alongside improvements in select markers of plasma antioxidant capacity [201]. Subsequently, beneficial effects on postprandial triglyceride responses were confirmed, which were abolished under acute psychological stress [202]. In a later trial employing more realistic culinary doses of a spice blend (2 g and 6 g), the lower dose reduced mean postprandial triglyceride levels, whereas no significant effects on glycemia or insulinemia were observed [204].

In adults with overweight or obesity, the cinnamon-containing meal reduced early postprandial glycemia, while both the cinnamon and pumpkin pie spice meals were associated with lower insulin concentrations at 30 min compared with the control meal, without significant differences in postprandial triglyceride concentrations [16].

Overall, available studies indicate that spices administered in food matrices may favorably modulate selected components of the postprandial response, particularly glycemia; however, these effects were not consistently observed across all metabolic endpoints. Several interventions showed no significant effects on postprandial glycemia, insulinemia, appetite-related outcomes, or energy metabolism, while findings regarding lipemic and appetite-regulating hormone responses remained less consistent. Evidence from clinically defined populations remains limited, and the small number of studies involving individuals with T2DM or overweight/obesity precludes meaningful comparisons between clinical subgroups. The only identified study directly comparing participants with T2DM and healthy controls indicated differential postprandial responses between these groups. The most reproducible beneficial effects have been observed in studies utilizing culinary spice blends, whereas results for individual spices were more variable and dependent on the specific spice and matrix used. Given that most available data derive from acute randomized crossover trials, the relevance of these observations for long-term metabolic regulation warrants further investigation.

#### 6.2.3. Vascular Function and Oxidative Stress

Existing studies regarding endothelial function and oxidative stress have almost exclusively utilized culinary spice blends added to high-fat meals, enabling the assessment of their potential impact on postprandial endothelial function and oxidative stress.

Initial studies demonstrated that adding an antioxidant spice blend to beef prior to thermal processing significantly limited malondialdehyde (MDA) formation during hamburger preparation. This was accompanied by lower postprandial plasma and urinary MDA concentrations in participants consuming the spice-supplemented hamburgers, suggesting that attenuating the generation of lipid peroxidation products during food preparation may decrease systemic postprandial exposure to these compounds [195].

The vascular relevance of culinary spice blends was subsequently demonstrated in men with type 2 diabetes. Adding the same spice blend to a high-fat hamburger attenuated postprandial endothelial dysfunction, as assessed by peripheral arterial tonometry, and reduced urinary MDA excretion. Urinary nitrate/nitrite concentrations were numerically higher after the spice-containing meal, although the difference did not reach conventional statistical significance [196].

Similar observations were obtained in a randomized crossover study using Japanese curry prepared with a spice blend. Single-dose ingestion of curry improved postprandial FMD compared to an isocaloric control meal, despite no significant changes in glucose, lipids, or oxidative stress markers. This suggests that the observed improvement in endothelial function may be mediated through mechanisms independent of classical postprandial metabolic changes [194].

Comparable findings were reported in a study conducted at Pennsylvania State University, in which the addition of 6 g of a culinary spice blend to a high-saturated-fat, high-carbohydrate meal attenuated the postprandial reduction in FMD compared to the spice-free meal, whereas the 2 g dose had no significant effect on FMD. These vascular effects were not accompanied by significant alterations in postprandial glucose or insulin responses [204].

Taken together, available research suggests that culinary spice blends may attenuate postprandial endothelial impairment and selected markers of oxidative stress, although these effects were not consistently observed across all doses and outcome measures. Favorable findings were most often reported for endothelial function and markers of lipid peroxidation. However, as the majority of current evidence stems from short-term intervention trials, the significance of these observations for long-term cardiovascular disease prevention requires further study.

#### 6.2.4. Other Emerging Research Areas

Beyond the metabolic effects described above, the impact of culinary spice blends on inflammatory responses and gut microbiota composition has also been evaluated. In the only randomized trial identified that specifically evaluated inflammatory outcomes, the highest tested dose of the spice blend reduced ex vivo IL-1β secretion and modified IL-8 and TNF-α secretion by LPS-stimulated peripheral blood mononuclear cells collected during the postprandial period, suggesting modulation of immune-cell responsiveness [203]. Notably, these effects were observed in cytokine secretion from LPS-stimulated PBMCs, whereas plasma cytokine concentrations were not significantly altered by spice consumption.

Within the Polyspice program, the influence of a culinary spice blend on gut microbiota composition was evaluated. Single-dose consumption of curry led to dose-dependent shifts in the relative abundance of select bacterial genera, including an increase in Bifidobacterium and a decrease in Bacteroides, without significant changes in alpha diversity. Furthermore, correlations were observed between shifts in microbiota composition and plasma concentrations of select phenolic metabolites, suggesting potential interactions between the metabolism of spice-derived bioactives and gut microorganisms [200].

## 7. Current Limitations and Future Research Directions

Despite the rapidly growing body of evidence suggesting potential beneficial effects of spice-derived phytochemicals, our understanding of how food matrices influence their biological activity remains incomplete. The available literature consistently indicates that food matrices are not passive carriers of bioactive compounds but active modulators of their stability, bioaccessibility, metabolism, and physiological effects. However, several important methodological and conceptual limitations currently hinder the translation of experimental findings into practical dietary recommendations.

An important distinction should be made between bioaccessibility, bioavailability, and bioactivity when interpreting the available evidence. In vitro digestion studies primarily provide information on the release, stability, and post-digestive availability of phytochemicals and therefore should not be considered direct evidence of systemic bioavailability. Animal studies provide additional information on absorption, metabolism, tissue distribution, and physiological effects, but their findings cannot be directly extrapolated to humans. Human intervention studies provide the most direct evidence of systemic exposure and physiological responses in humans; however, the currently available trials are predominantly acute and provide limited evidence regarding long-term health outcomes. Accordingly, increased bioaccessibility observed in vitro should not automatically be interpreted as increased systemic bioavailability or clinical efficacy.

One of the major limitations of the existing literature is the predominance of in vitro studies. Simulated gastrointestinal digestion models have substantially improved our understanding of matrix-dependent changes in the stability and bioaccessibility of spice-derived phytochemicals, allowing systematic comparisons between different food systems under controlled conditions. Nevertheless, these models cannot fully reproduce the complexity of human digestion, including dynamic gastrointestinal physiology, interindividual variability, intestinal absorption, first-pass metabolism, tissue distribution, interactions with the gut microbiota, and long-term physiological adaptation. Although animal studies partially bridge this gap, species-specific differences in digestive physiology and metabolism limit the direct extrapolation of their findings to humans.

Human intervention studies remain relatively scarce and are characterized by considerable heterogeneity. Most available clinical trials have investigated acute postprandial responses using randomized crossover designs in healthy adults, whereas long-term interventions evaluating habitual consumption of spice-enriched foods are still uncommon. Furthermore, relatively few studies have included individuals with obesity, metabolic syndrome, type 2 diabetes, or other chronic diseases that are most likely to benefit from increased dietary intake of spice-derived bioactive compounds. Consequently, the long-term clinical relevance of the observed improvements in postprandial metabolism, oxidative stress, endothelial function, and inflammatory responses remains insufficiently established.

Another important challenge is the substantial methodological variability among studies. Considerable differences exist in the botanical origin of spices, extraction procedures, processing conditions, encapsulation technologies, doses of bioactive compounds, food matrices, storage conditions, and analytical methods. These differences complicate direct comparisons between studies and limit the possibility of performing robust quantitative syntheses or meta-analyses. Greater standardization of experimental protocols, including the use of unified in vitro digestion models, standardized spice extracts, and validated analytical methods, would considerably improve the comparability and reproducibility of future research.

The analytical determination of polyphenols represents another important limitation. Widely used colorimetric methods, particularly the Folin–Ciocalteu assay, are susceptible to interference from proteins, peptides, amino acids, reducing sugars, and other phenolic-like compounds present in food matrices, as well as undetectable polyphenols in protein complexes. Consequently, changes in measured total phenolic content do not always reflect actual changes in polyphenol concentration or bioavailability. Future studies should increasingly combine conventional spectrophotometric assays with advanced chromatographic and mass spectrometric techniques capable of identifying individual metabolites and monitoring their transformation throughout digestion and metabolism.

The current evidence is also highly uneven among different spices. Most mechanistic and clinical studies have focused on turmeric and curcuminoids, largely because of their inherently low bioavailability and the considerable potential for matrix-dependent improvement. In comparison, substantially fewer studies have investigated ginger or cinnamon, while evidence concerning cloves remains severely limited despite their exceptionally high polyphenol content and antioxidant potential. Similar disparities exist regarding food matrices, with dairy products and cereal-based foods receiving considerably greater attention than meat products, plant-based dairy alternatives, fermented plant foods, or mixed meals representative of habitual dietary patterns. Therefore, there is a clear need to extend research to a wider range of spices and their bioactive compounds in the context of interactions with a diverse food matrix.

Although numerous studies demonstrate that proteins, lipids, polysaccharides (including dietary fiber), sweeteners, vitamins, and minerals, as well as food processing (e.g., fermentation) modify the biological activity of spice-derived phytochemicals, the underlying molecular mechanisms remain only partially understood. In particular, the physicochemical interactions between phytochemicals and food macromolecules, including protein–polyphenol, lipid–polyphenol, and polysaccharide–polyphenol complexes, require further investigation using modern structural and molecular approaches. A better understanding of these interactions would facilitate the rational design of functional foods that maximize the stability and bioavailability of spice-derived bioactive compounds while maintaining desirable technological and sensory properties.

Another rapidly developing research area concerns the role of the gut microbiota in mediating matrix-dependent effects of spice phytochemicals. Increasing evidence suggests that gastrointestinal microorganisms actively transform complex polyphenols into lower-molecular-weight metabolites with improved bioavailability and potentially greater biological activity. Conversely, food matrices may modify microbial composition and metabolic activity, thereby influencing both the generation of bioactive metabolites and host responses. Future studies integrating microbiome analysis with metabolomics and controlled dietary interventions are needed to clarify these complex bidirectional interactions.

Relatively little attention has also been devoted to interactions between different spices within complex culinary mixtures. Available evidence indicates that spice combinations may produce synergistic, additive, or antagonistic effects depending on their chemical composition and the surrounding food matrix. Since spices are typically consumed as components of mixed meals rather than as isolated ingredients, further research should investigate realistic culinary formulations and identify combinations that maximize health-promoting properties while preserving product quality and consumer acceptability.

Future research should also move beyond evaluating isolated bioactive compounds toward investigating whole foods and dietary patterns. The health effects of spice-derived phytochemicals are likely to depend not only on the characteristics of individual food matrices but also on interactions occurring within complete meals consumed under habitual dietary conditions. Such studies would provide greater ecological validity and facilitate the translation of experimental findings into evidence-based nutritional recommendations.

Finally, advances in food technology offer promising opportunities for improving the delivery of spice-derived phytochemicals. Novel encapsulation systems, nanoemulsions, protein- and polysaccharide-based carriers, Pickering emulsions, and other clean-label delivery systems have the potential to enhance compound stability, control gastrointestinal release, and increase bioavailability while minimizing undesirable changes in food quality.

Future research should adopt multidisciplinary approaches integrating food chemistry, food technology, gastrointestinal physiology, microbiology, metabolomics and clinical nutrition. Such an integrated strategy will be essential for elucidating the complex interactions between spice-derived phytochemicals and food matrices and for translating mechanistic knowledge into the rational design of functional foods with scientifically substantiated health benefits.

## 8. Conclusions

The available evidence suggests that the food matrix can substantially influence the stability, bioaccessibility, metabolism, and biological effects of spice-derived phytochemicals. These effects appear to result from interactions with proteins, lipids, carbohydrates, dietary fiber, vitamins, minerals, and the gut microbiota. However, much of the evidence supporting these mechanisms derives from in vitro and animal studies, whereas confirmation in human intervention trials remains limited and heterogeneous.

Among the investigated matrices, dairy products and lipid-rich systems appear to be particularly effective carriers of lipophilic compounds such as curcuminoids, whereas cereal-based products enriched with spices may provide additional benefits through increased dietary fiber and improved glycemic properties. Food processing, extraction methods, encapsulation technologies, and the composition of mixed meals further influence the release and biological efficacy of spice-derived phytochemicals. However, matrix effects are not universally beneficial. High concentrations of proteins, dietary fiber, or other macromolecules may promote phytochemical binding, reduce their release from the food matrix, or delay their gastrointestinal availability. Therefore, the relationship between matrix composition and phytochemical bioaccessibility or bioactivity may be nonlinear and dose-dependent, with the same matrix component potentially enhancing stability or protection under some conditions while limiting phytochemical release or activity under others. Moreover, the available evidence indicates that interactions between different spices may be either synergistic or antagonistic, emphasizing the importance of considering the entire food matrix rather than isolated compounds.

Although numerous in vitro and animal studies provide substantial mechanistic evidence, human intervention studies remain limited and are largely restricted to acute postprandial responses. Consequently, further well-designed, long-term clinical trials using standardized food matrices and analytically characterized spice preparations are required to establish dose–response relationships and determine whether these effects translate into long-term health benefits of spice-enriched foods, in both healthy and diseased individuals.

From a practical perspective, the findings summarized in this review indicate that the development of spice-enriched functional foods should consider the food matrix, processing conditions, and the form and dose of spice supplementation as integral components of product design. For lipid-soluble phytochemicals such as curcuminoids, dairy- or lipid-containing matrices may provide favorable conditions for solubilization and gastrointestinal availability, whereas cereal-based products may be particularly suitable when the aim is to increase dietary fiber and improve glycemic properties. In meat products, antioxidant spices such as ginger may contribute to oxidative and microbiological stability, while in beverages the choice of sweetener, protein or lipid components, and fermentation conditions may influence phytochemical stability and bioactivity. In addition, combinations of aromatic and spicy plants should be selected on the basis of their potential synergistic or complementary effects, while considering the possibility of antagonistic interactions. Thus, optimization of the spice–matrix combination, dose, processing conditions, and delivery form should be tailored to the specific food category and the targeted biological effect.

Overall, current findings support further investigation of the food matrix as an important factor influencing the biological effects of spice-derived phytochemicals. However, the available human evidence does not yet permit firm conclusions regarding sustained health benefits of spice-enriched foods, and matrix-dependent effects observed in preclinical and acute human studies should not yet be translated into evidence-based dietary recommendations.

## Figures and Tables

**Table 1:** Major classes of bioactive compounds present in spices, their main sources, and biological activities.

Spice	Main Bioactive Compounds	Main Bioactive Activity	Health Benefits
Cinnamon (*Cinnamomum*)	Hydroxycoumarins cinnamaldehyde, cinnamic acid, cinnamate, limonene, eugenol, terpineol, catechins, tannins, linalool, proanthocyanidins, safrole, pinene, methyleugenol, benzaldehyde	Antibacterial, antimicrobial, antifungal, antiviral, antioxidant, anti-inflammatory	↑ coronary blood flow, cardiac contractile force, insulin sensitivity, GLP-1, GSH, SOD; ↓ peripheral vascular resistance, LDL-C, FBG, postprandial glucose, postprandial insulin, HbA1c, HOMA-IR, TC, LDL-C, TG, ALT, AST, GGT, CRP, MDA, SBP, DBP, BMI, WHR, fat mass, visceral fat
Turmeric (*Curcuma longa* L.)	Curcuminoids, bisdemethoxycurcumin, tetrahydrocurcumin, demethoxycurcumin, carotene, capsaicin, eugenol, ascorbic acid, caffeic acid, *p*-coumaric acid, Protocatechuic acid, vanillic acid, syringic acid	Antimicrobial, antibacterial, anti-inflammatory, antioxidant, antitumor	↓ TG, TC, LDL-C, arterial stiffness, thromboxane platelet aggregation, cardiomyocytic apoptosis, FBG, serum insulin, HOMA-IR, leptin, WC, BP, MetS score, AST, ALT, GGT, inflammatory markers, BMI; improvement in body composition; ↑ HDL-C, GSH
Clove (*Syzygium aromaticum*)	Eugenol, isoeugenol, dehydroeugenol, caryophyllene, sesquiterpenes, tannins, acetyleugenol pinene, gallic acid, vanillin, flavonoids, phenolic acids	Antibacterial, antimicrobial, antifungal, anticancer, antioxidant, anti-inflammatory, analgesic	↑ HDL-C; ↓ LDL-C, TG, insulin resistance, hypertension, TNF-α, IL-6
Ginger (*Zingiber officinale*)	Gingerol, shogaols, paradol, zingerone, hydoxyphenylpropenes, vallinoids, borneol, linalool, geraniol, geranial, camphene, zingerol, zingiberon	Antibacterial, antimicrobial, antiviral, antitumor, anti-inflammatory, antioxidant, antiplatelet formation	↓ LDL-C, TC, ApoB, atherogenic modifications, platelet aggregation, oxidative response of macrophages; CRP, MDA, TNF-α, AST, FBG, HbA1c, serum insulin, insulin sensitivity, HOMA-IR, BMI, WC, appetite; ↑ HDL-C, QUICKI

Source: prepared based on [18,19,20]. Abbreviations: GLP-1, glukagone-like peptide-1; GSH, glutathione; SOD, superoxide dismutase; LDL-C, low-density lipoprotein cholesterol; FBG, fasting blood glucose; HbA1c, glycated hemoglobin; HOMA-IR, homeostatic model assessment for insulin resistance; TC, total cholesterol; TG, triglycerides; ALT, alanine aminotransferase; AST, aspartate aminotransferase; GGT, gamma-glutamyltransferase; CRP, *C*-reactive protein; MDA, malondialdehyde; SBP, systolic blood pressure; DBP, diastolic blood pressure; BMI, body mass index; WHR, waist–hip ratio; WC, waist circumference; BP, blood pressure; MetS, metabolic syndrome; HDL-C, high-density lipoprotein cholesterol; TNF-α, tumor necrosis factor alpha; IL-6, interleukin-6; ApoB, apolipoprotein B; QUICKI, quantitative insulin sensitivity check index; ↓, decrease; ↑, increase.

**Table 2:** Influence of food matrices on the biological effects of selected spices in in vitro studies of functional foods.

Spice	Food Matrix	Dose	Study Design	Main Biological Effects	Ref.
Cinnamon	Wheat flour-based bread	0.5%, and 1% of cinnamon powder	Control: 100% wheat flour-based bread without cinnamon	↑ protein, fat, fiber, and mineral content, TPC, DPPH; high sensory acceptance (for 0.5%); ↓ TFC (for 0.5%), carbohydrates content; low sensory acceptance (for 1%)	[105]
	White wheat bread (baguette)	0.25–1% (*w*/*w*) of cinnamon powder	Control: white wheat bread without spices	↓ furan derivatives content, 2-pentyl furan content; ↑ DPPH, fiber content	[106]
	Oil cake	0.05–0.25% (*w*/*v*) aqueous cinnamon bark extract	Control: oil cake without spices	↑ TPC, TTC, RSA, and FRAP, protein, fiber, and minerals content; ↓ fat and carbohydrates content; high sensory acceptance (for 0.2%, 0.25%)	[107]
	Traditional cakes	0.06–0.24% (*w*/*w*) of cinnamon powder	Control: Traditional cakes without spices	↑ acrylamide content (dose dependent); acceptable THQ and ILCR	[108]
	Fermented milk (FM)	20% (*w*/*v*) bark (CB) and twig (CT) aqueous extracts	Control: fermented milk without extracts (0%); FM with free (E) vs. encapsulated (EE) forms extracts; lactic fermentation; static in vitro digestion model	Pre-digestion: ↑ TPC, DPPH, FRAP (CB > CT) ↑ fermentation (↑ acidity, ↓ sugar content) Post-digestion: ↓ TPC, TFC, DPPH, FRAP, excluding E-CB-FM)	[109]
	Stirred yogurt	0.8% (*w*/*v*) aqueous cinnamon leaf extract (CLE)	Control: plain stirred yogurt (0%); free (E) vs. encapsulated (EE) extracts; static in vitro digestion model	Pre-digestion: ↑ TPC, TFC, DPPH and FRAP; ↓ fermentation process (E/EE vs. control); ↑ pH; ↓ synerase, no ADI (EE vs. control) Post-digestion: ↓TFC, DPPH, FRAP (E/EE = control), although lighter effect for EE; ↑ ADI in EE	[110]
	Stirred yogurt	Y + SLN: 0.02% (*w*/*w*) of curcumin added in SLN form	control: (1) Y: plain stirred yogurt without SLN (2) pure SLN (0.1% curcumin); dynamic in vitro digestion model (DIVGIS system)	Pre-digestion: ↑ organoleptic and technologic characteristics (Y + SLN) Post-digestion: no negative effect for BI and stability of curcumin: (Y + SLN = SLN); ↓ protein solubility index: Y + SLN < Y; cytotoxic effect (MTT: Y + SLN 10.3% vs. Y or SLN > 80%)	[111]
	Yogurt	0.1–0.3% aqueous cinnamon extract	Control: plain yogurt without cinnamon	↑ TPC, TFC, tannin content, DPPH	[112]
	Cow’s milk yogurt	1% ethanol extract of cinnamon (C)	Other variants: 1% ethanol extract of Fenugreek (F) or Aloe vera (A); mixed variants (C-F, C-A, F-A)	The highest α-glucosidase inhibition (anti-diabetic effect) in cinnamon version; mixed versions with no activity	[113]
	Yogurt	0.5–1.5% of cinnamon extract	Control: plain yogurt without spice extract	↑ TPC, DPPH	[114]
	Tiger nut yogurt (plant-based)	0.5–2% of cinnamon extract	Control: plain yogurt made from tiger nut milk without cinnamon	↑ mineral content (P, K, Na, Fe, Zn)	[115]
	Vegan white chocolate with soy protein and/or coconut flour	2% (*w*/*w*) encapsulated cinnamon extract (ECE)	Control: vegan white chocolate with soy protein and/or coconut flour in different share without ECE	↑ TPC, TFC, DPPH (irrespectively of soy protein and/or coconut flour share)	[116]
	Traditional Egyptian cinnamon beverage (TECB)	TECB: 2 g of cinnamon bark powder per 100 mL of boiled water	Control: plain TECB; other versions: TECB with sucrose (S): 2.5 g/100 mL, TECB with acacia honey (H): 5 g/100 mL, TEBC with cow’s milk: 25% *v*/*v*; static in vitro digestion model	Pre-digestion: high TPC, ABTS (TECB); no negative effect of sweeteners for TPC Postdigestion: no negative effect of milk on BI (TECB= TECB with milk) ↑ BI by sweeteners (S > H) in TECB; milk neutralizes this effect; ↑ ABTS of TECB by milk	[117]
	Soursop (*Annona muricata*) leaf tea	10–30% of cinnamon powder	Control: Soursop leaf tea without cinnamon	↑ DPPH	[118]
Ginger	Wheat bread	2–8% ginger powder incorporated into white flour	Control: 0% ginger powder incorporated into white flour	↑ TPC, DPPH ↑ mineral content (Na, Ca, K, Fe, Zn, Cu)	[119]
	Wheat cookies	5–15% ginger powder	Control: wheat cookies	↑ TPC, DPPH, ABTS ↑ energy value, protein, fat, fiber, ash content	[120]
	Cookies (from wheat flour)	ginger powder in concentrations of 2–14% to wheat flour	Control: Cookies (from 100% wheat flour)	↑ TPC, DPPH, fiber, mineral content	[121]
	Biscuits	1–7% (*w*/*w*) ground ginger powder	Control: biscuits without ginger	↓ acrylamide content (dose-dependent)	[122]
	Yogurt	0.5–1.5% of ginger extract	Control: plain yogurt without spice extract	↑ TPC, DPPH	[114]
	Yogurt (fermented bovine milk)	0.5–2.5% (*w*/*v*) ginger powder	Control: plain yogurt without ginger	↑ TPC, DPPH ↑ protein and fat content	[123]
	Ice cream	Ginger Juice: 2–8%, Ginger Paste: 2–8%, Ginger Candy: 5–20%, Ginger Powder: 0.5–2.0%	Control: plain ice cream (standard plain mix with 11% fat and 11% SNF) without ginger	↑ TPC, ABTS (dose-dependent, irrespective of the form)	[124]
	Robusta coffee (*Coffea canephora*)	ginger extract to coffee extract: 10–20 mL:50 mL and 10–20 mL:40 mL	decaffeinated (D) and non-decaffeinated coffee (ND)	↑ TPC, DPPH (ND > D)	[125]
	Pork burgers	BG1: Meat added with 1% ginger powder (10 g/kg) BG2: Meat added with 2% ginger powder (20 g/kg)	Control: plain pork burgers without ginger	↑ ABTS, FRAP, PUFA, ω3/ω6 ratio ↓ TBARS, SFA, AI, TI, h/H ratio BG2 > BG1 > control	[126]
	Rabbit burgers	Z1: Meat supplemented with 1% ginger powder. Z2: Meat supplemented with 2% ginger powder	Control: plain rabbit burgers without ginger; analyzed at day 1, 4, and 7 (storage at 4 °C) in both raw and cooked forms	↑ ABTS, FRAP, PUFA, ω3/ω6 ratio ↓ TBARS, AI, TI, h/H ratio Z2 > Z1 > control	[127]
	Various homogenized diets: high-fiber diet (FD), basic diet (BD), standard diet (SD)	0.2 g of concentrated ginger extract mixed with 2 g of diet FD 50.3 g fiber, BD 27.8 g fiber, SD 21.0 g fiber; FD with the highest protein, fat, vitamins and minerals content	Control: 0.2 g of concentrated ginger extract mixed with 2 mL of water; static in vitro digestion model	Post-digestion: ↓ ginger PP by 55–99%; ↓ gingerPP bioavailability in all diets and control; bioavailability: control > FD> SD > BD;	[100]
Turmeric	Biscuits (B) and custards (C)	Custards enriched with 35.6 mg/100 g and biscuits with 106.7 mg/100 g of curcuminoid powder (curcumin content approximately 75%)	Biscuits and custards with 9 different fiber sources (inulin, maize fiber, carrot fiber, apple fiber, yellow pea fiber, wheat fiber, and microcrystalline cellulose); 2 matrix types (liquid-like custard vs. solid biscuit); static in vitro digestion model	Pre-digestion: heat-resistance of curcuminoids (160 °C); Post-digestion: ↑ BI by dietary fiber (excluding cellulose), especially by soluble fiber and hemicellulose; ↓ BI by cellulose; bioaccessibility B > C (due to higher fat and sugar content); low BI for water/milk	[104]
	Muffins	M1 (2.5% turmeric flour), M2 (5% turmeric flour), and M3 (10% turmeric flour)	Control: muffin (100% wheat flour); 14-day storage period; static in vitro digestion model	Pre-digestion: ↑ TPC, TFC, DPPH, ABTS, minerals (ash), fiber; Post-digestion: ↓ *α*-amylase and *α*-glucosidase enzymes, IC_50_, eGI, IVSD, MDA (dose-dependent)	[103]
	UHT milk	formula 1: 0.9% turmeric powder; formula 2: 1.9% turmeric powder; formula 3: 2.8% turmeric powder	Control: plain commercial full cream UHT milk	↑ curcumin, DPPH ↓ hedonic value (dose-dependent)	[128]
	Skimmed milks (3.7% protein and 5% fat)	curcumin nanoemulsion (soybean oil, 0.03% curcumin)	Milks reconstituted from low-heat (MLH), medium-heat (MMH), and high-heat (MHH) skim milk powders; dynamic in vitro digestion model	Post-digestion: high BI of curcumin (>70%) in all variants; for MHH the highest BI of curcumin, release of FFA, and casein hydrolysis; ↓ curcumin release efficiency for MLH/MMH	[129]
	Skimmed-milk yogurt and UF-Kariesh cheese	10% *w*/*w* aqueous milled turmeric extract at 1% in yogurt and 2% in cheese	Control: skimmed-milk yogurt and UF-Kariesh cheese without spices; lactic fermentation	↑ TPC, TFC, DPPH (cheese > in yogurt); ↑ LAB	[130]
	Yogurt	0.5% (TP_05) and 1.0% (TP_1) of turmeric powder (TP)	Control: plain yogurt; static in vitro digestion model	Pre-digestion: ↑ TPC, DPPH; ↓ synerase; ↓ sensory acceptance; ↓ yogurt bacteria load (dose-dependent), although acceptable; ↑ pH; Post-digestion: ↓ PP, DPPH (TP_1) PP andDPPH for TP_05/TP_1 > control	[13]
	Fermented dairy product with plantain (*Musa paradisiaca*) syrup (50% *w*/*w*)	Turmeric powder at 0.1–0.5% (*w*/*w*)	Control: fermented dairy product with plantain (*Musa paradisiaca*) syrup (50% *w*/*w*) without turmeric	↑ TPC, Ca content ↓ IC_50_, TBARS Dose-dependent	[131]
	Lassi (a traditional Indian fermented cow milk beverage, 3% fat)	1–4% turmeric extract	Control: plain lassi without turmeric	↑ TPC; the highest sensory acceptance (for 1%); lassi as an excellent vehicle for lipophilic curcumin	[132]
	Kombucha	0.4–2% turmeric powder in 500 mL boiling water (TK)	Control: 0.4% Black Tea Kombucha; turmeric extracts were fermented with an SCOBY (Symbiotic Culture of Bacteria and Yeast) and 10% sugar for 12 days at room temperature	↑ TPC during fermentation; E. coli antibacterial effect (TK > control) ↓ fermentation, microbial load, TPC (for >0.8%); optimal turmeric addition: 0.8%	[101]
Clove	Yogurt	4% clove extract	Control: plain yogurt without clove	↑ TPC, TFC, TAC, DPPH, FRAP, NO scavenging, MDA	[133]
Cinnamon, Garlic, Ginger	Cow milk yogurt	cinnamon bark extract 0.5–1.0% (C), garlic extract 0.5–1.0%, ginger extract 0.5–1.0% (G)	Control: plain cow milk yogurt without spices	↑ vitamin A (C, G), Ca (C), protein (1% C, G), fat (C, G) ↑ IC_50_, DPPH	[134]
Cinnamon, Ginger	Air-dried pumpkin chips (PC)	1% of Ginger or cinnamon powder	Control: PC without spices; PC with ginger (PCG) vs. PC with cinnamon (PCC); static in vitro digestion	Pre-digestion: ↑ TPC, ABTS, DPPH, FRAP, CUPRAC vs. control (PCC > PCG); Post-digestion: ↑ BI by 50% (PCC > PCG); AA: PCC > PCG > PC	[135]
Turmeric, Saffron	Muhallebi (traditional turkisch milk pudding)	Saffron powder 0.02% Turmeric powder 0.02% Mixed (0.02% saffron + 0.02% turmeric)	Control: plain Muhallebi without spices	↑ TPC, ABTS, TEAC Mixed variant: the highest TPC, ABTS, TEAC	[136]
Cinnamon, Ginger	Roselle (*Hibiscus sabdariffa*) beverage (R)	1 g/L of ground cinnamon bark (RC) or 1 g/L of ground ginger (RG)	Control: unsweetened Roselle beverage (uR); R + sucrose 80 g/L (Rsu); R + stevia 0.32 g/L (Rst); uRC; RCsc; RCst; uRG; RGsu; RGst	RC: ↑ FRAP, t_1/2_ at 40 °C from 7 to 13 days; ↓ anthocyanin degradation; RCsu: ↑ t_1/2_ at 40 °C from 7 to 21 days, no protective effect of giger (t_1/2_ 5–6 days)	[137]
Ginger, Sappan wood, Turmeric, Cinnamon, Clove, Nutmeg	Wedang Uwuh (traditional Indonesian Beverage)	Ginger 56.1%, Sappan wood 19.6%, Clove stems 7.3%, Nutmeg leaves 6.1%, Cinnamon leaves 6.0%, Clove leaves 4.9% in hot water	No control; boiling time R5, R10, R15 (5, 10, 15 min); static in vitro digestion	Pre-digestion: TPC, FRAP, DPPH (R15 > R10 > R5); Post-digestion: ↓ TPC, FRAP, DPPH, ABTS, irrespectively of boiling time	[138]

Abbreviations: *w*/*w*, weight/weight; *w*/*v*, weight/volume; TPC, total phenolic content; DPPH, 2,2-difenylo-1-pikrylohydrazyl scavenging activity; TFC, total flavonoid content; TTC, total tocopherol compounds; RSA, radical scavenging activity; FRAP, ferric reducing antioxidant power; THQ, Target Hazard Quotient; ILCR, Incremental Lifetime Cancer Risk; ADI, albumin denaturation inhibition; SLN, solid lipid nanoparticles; BI, bioaccessibility index; MTT, metabolic tolerance test; P, phosphorus; K, potassium; Na, sodium; Fe, iron; Zn, zinc; ABTS, 2,2′-azino-bis- (3-ethylbenzothiazoline-6-sulfonic) acid scavenging activity; Ca, calcium; Cu, copper; SNF, solid-not-fat; PUFA, polyunsaturated fatty acids; TBARS, thiobarbituric acid reactive substances; SFA, saturated fatty acids; AI, Atherogenicity Index; TI, Thrombogenicity Index; h/H ratio, hypocholesterolemic/hypercholesterolemic ratio; PP, polyphenols; IC_50_, half maximal inhibitory concentration; eGI, estimated glycemic index; IVSD, in vitro starch digestibility; MDA, malondialdehyde; FFA, free fatty acid; UF, ultrafultered; LAB, lactic bacteria; TAC, total antioxidant capacity; NO, nitric oxide radical; CUPRAC, cupric ion reducing antioxidant capacity; AA, antioxidant activity; TEAC, Trolox equivalent antioxidant capacity; t_1/2_, half life; ↓, decrease; ↑, increase.

**Table 3:** Influence of food matrices on the biological effects of selected spices and spice-derived bioactives in in vivo animal studies.

Spice	Food Matrix	Dose	Study Design	Main Health and Nutritional Effects	Ref.
Turmeric	Natural dairy yogurt; protein–lipid matrix (Nestlé)	30, 60, 90 mg/kg BW/day; curcumin; oral (gavage)	Wistar rats (STZ-diabetic), n = 8/group, 31 days. Control: water, plain yogurt, insulin	↓ glycemia, TAG, and liver enzymes (ALT, AST); ↑ BW	[172]
	Corn-soybean diet (plant-based feed)	200–300 mg/kg feed; curcumin; ad libitum	Wuzhishan piglets (weaned), n = 10/group, 21 days. Control: basal diet	Improved intestinal morphology and barrier function; ↑ serum SOD activity	[17]
	High-fat diet (HFD)- 50% of the calories derived from fat; lipid-rich matrix	20 mg/kg; curcumin degradants (FER/DFER); oral	Mice (HFD-induced obesity). Control: standard diet, n = 10/group, 16 weeks	Prevention of weight gain and fatty liver; ↑ SCFA levels in the gut	[173]
	Aqueous suspension with essential oil	25–50 mg/kg/day; curcuminoids; oral	Mice (AlCl3-induced neurotoxicity), n = 6, 45 days. Control: vehicle	Neuroprotection of hippocampal cells; improved memory and learning parameters	[174]
Ginger	HFHF diet (High-fat high-fructose)	50, 100, 200 mg/kg; 6-gingerol; oral (gavage)	Sprague-Dawley rats, n = 5/group, 8 weeks. Control: standard diet	↓ liver steatosis (NAFLD); ↓ r ER stress markers and p-NF-κB	[175]
	High-fat diet (HFD)	Aza-6-gingerol (A6G)-0.06% (stable analog); diet supplementation	Mice (obesity/T2DM model), n = 5/group, 86 days. Control: regular chow	↓ body fat accumulation, insulin, and leptin; inhibited lipogenesis	[176]
Cinnamon	Defatted soy flour (CDSF); plant protein matrix	600 mg/kg CDSF (cinnamon extract); oral	Mice (VHFD-induced obesity), n = 8–15, 5 h (acute study). Control: plain soy flour	↓ FBG 6 h after administration	[177]
	Drinkable yoghurt; microencapsulated in alginate	5, 10, 20 mg flavonoids/kg; oral	Rabbits (Metabolic Syndrome model). Control: untreated MetS group	↓ glucose, TC, and LDL; ↓ in abdominal girth	[178]
	Camel milk yoghurt;	23 mg/kg (extract)	Albino Wistar rats (STZ-diabetic), n = 9/group, 21 days. Control: milk, insulin	↓ in serum glucose, TC and TAG	[179]
	High-fat diet (HFD)	40 mg/kg/day; cinnamaldehyde; oral (gavage)	Mice (C57BL/6J), n = 8/group, 8 weeks. Control: standard diet	Induction of WAT browning; ↓ fat mass and improved insulin sensitivity	[180]
	Oatmeal cookies	Ad libitum in diet; cinnamon powder (5%)	Rats with liver damage (CCl4), n = 5/group, 28 days. Control: standard diet	Positive effect on the regeneration of liver and kidney tissues	[181]
	HFS diet (High-fat high-sucrose)	0.1–1.0% cinnamaldehyde in diet	Mice (obesity model C57Bl/6 Cr), n = 6/group, 30 days. Control: HFS diet without additive	↓ visceral fat; ↓ BW	[182]
Clove	High-fat diet (HFD)	0.2% eugenol (*w*/*w*) in diet	C57BL/6J mice, 13 weeks	↓ body weight gain, ↓ visceral adiposity, ↓ fasting glucose; modulation of gut microbiota; improved adipose tissue transcriptome	[183]
	Standard diet + clove supplementation	equivalent to 100 mg eugenol + eugenyl acetate/kg BW/day	STZ-diabetic Sprague-Dawley rats	Protection of heart, liver and lens against diabetic damage; reduced tissue injury	[184]
	Feed supplementation	essential oil in finishing diet	Nellore heifers	No major metabolic effects, altered rumination behaviour and feed utilization	[185]
Turmeric Black Pepper	Natural yoghurt	90 mg/kg curcumin + 20/40 mg/kg piperine; oral	Wistar rats (STZ-diabetic), n = 6/group, 45 days. Control: yoghurt, insulin	40 mg/kg piperine nullified the antidiabetic and antioxidant benefits of curcumin	[186]
Thyme Echinacea Oregano Cinnamon	TMR ration; maize and grass silage matrix	3% of concentrate dry matter	Holstein-Friesian cows, n = 15/group, 6 weeks. Control: standard TMR	↑ casein, lactoferrin, and vitamins A/E in milk; ↑ antioxidant potential	[187]
Turmeric Black Pepper	0.5% CMC suspension	ED50 ratio 1:1; curcumin + piperine; oral	ICR mice, n = 8–10/group, 5–8 weeks. acute study. Control: 0.5% CMC	Strong synergistic analgesic effect in pain tests	[188]

Abbreviations: STZ, streptozotocin; TAG, triacylglycerols; ALT, alanine aminotransferase; AST, aspartate aminotransferase; BW, body weight; SOD, superoxide dismutase; HFD, high-fat diet; FER, feruloyl acetone; DFER, demethoxyferuloyl acetone; SCFA, short-chain fatty acids; AlCl3, aluminum chloride; HFHF, high-fat high-fructose; NAFLD, non-alcoholic fatty liver disease; ER stress, endoplasmic reticulum stress; p-NF-κB, phosphorylated nuclear factor kappa B; T2DM, type 2 diabetes mellitus; CDSF, defatted soy flour; VHFD, very high-fat diet; FBG, fasting blood glucose; MetS, metabolic syndrome; TC, total cholesterol; LDL, low-density lipoprotein; WAT, white adipose tissue; CCl4, carbon tetrachloride; HFS, high-fat high-sucrose; *w*/*w*, weight/weight; TMR, total mixed ration; CMC, carboxymethyl cellulose; ED50, median effective dose; ICR, Institute of Cancer Research; ↑, increase/improvement; ↓, decrease/worsening.

**Table 4:** Characteristics of human intervention studies investigating spices and spice-derived compounds administered in food matrices.

**Single Spice Interventions (Turmeric/Curcumin, Ginger and Cinnamon)**					
**Spice/Bioactive Compound**	**Food Matrix/Dietary Intervention**	**Dose and Mode** **of Administration**	**Study Design**	**Main Findings**	**Ref.**
Curcuminoids	Mango nectar, sports nutrition bar, oat drink used as a dairy analogue, pectin gummies and probiotic drink; capsule reference	300 mg Turmipure Gold^®^ per intervention, providing approximately 80–101 mg measured curcuminoids; incorporated into 60 mL mango nectar, 32 g sports bar, 240 mL oat drink, 10 g gummies or 100 g probiotic drink; 300 mg capsule as reference	Randomized, open-label, six-period crossover; healthy adults completing the study (n = 35); 24 h pharmacokinetic study	Dairy analogue, sports nutrition bar and probiotic drink: ↑ dose-normalized AUC24h and Cmax vs. capsules; ready-to-drink nectar and gummies: ↔ bioavailability vs. capsules.	[12]
Turmeric Curcumin	Mashed potato with cream; matched control meal without turmeric or curcumin	Fresh turmeric, turmeric powder or food-grade isolated curcumin incorporated into 200 g mashed potato and 108 mL cream; each active meal provided 400 mg curcumin	Randomized, four-condition crossover; healthy men (n = 13–14 analysed per condition); 6 h pharmacokinetic study	↑ Plasma curcumin concentrations at 1–3 h after fresh turmeric and turmeric powder vs. isolated curcumin; turmeric powder produced the highest curcumin AUC and Cmax and ↑ plasma DMC and BDMC concentrations	[11]
Curcuminoids	Curcumin-enriched bread	100 g bread containing 1 g total curcuminoids as free curcumin, microencapsulated curcumin or microencapsulated curcumin combined with piperine, quercetin and genistein; approximately 0.8 g curcumin per portion	Randomized, single-blind, three-period crossover; healthy adults (n = 10); 24 h bioavailability study	↑ Serum curcuminoid bioavailability (higher Cmax and delayed absorption with microencapsulation); ↑ phenolic acid metabolites (especially ECBB); ↑ overnight urinary curcuminoid excretion; ↑ urinary glucuronide metabolites (ECBB); ↑ fecal curcuminoids (ECB) and phenolic acids (ECBB)	[10]
Turmeric	Turmeric-based beverage consumed 10 min before an isoenergetic medium-fat or high-fat breakfast	220 mL turmeric beverage containing 185 mg GAE; matched control beverage; test meals provided 423 kcal	Randomized crossover; healthy adults (n = 12; 5 men and 7 women); acute postprandial study	↔ postprandial hunger, desire to eat, satiety, fullness and prospective consumption after either medium- or high-fat meal	[189]
Ginger Mustard, Horseradish, Black pepper	Standardized brunch; ginger incorporated into the stewed-apple component	20 g finely chopped fresh ginger; comparison arms contained 21 g Dijon mustard, 8.3 g horseradish, 1.3 g black pepper or no spice	Randomized, placebo-controlled, single-blind, five-period crossover; healthy normal-weight men (n = 22); acute postprandial study	↔ diet-induced thermogenesis, subjective appetite, subsequent ad libitum energy intake, energy balance and hemodynamic factors after ginger	[190]
Cinnamon	Raw cinnamon powder mixed with water or hard gelatin capsules consumed immediately before a standardized breakfast	3 or 6 g raw cinnamon powder mixed with 150 mL water or administered in hard gelatin capsules; followed immediately by a nutritionally balanced breakfast providing 50 g complex carbohydrate; control breakfast without cinnamon	Randomized, five-condition crossover; men with T2DM (n = 19); acute postprandial study (2 h)	Cinnamon suspension: ↓ postprandial blood glucose peak, Δ1-h glycemia and glucose iAUC vs. capsules; ↔ 2 h glucose concentration and time-to-peak	[14]
Turmeric Cinnamon	Dairy yogurt	Cinnamon or turmeric oleoresin incorporated separately into yogurt at a concentration of 1.0 × 10^−4^; oleoresins contained 30% spice extract	Randomized crossover; healthy adults (n = 16); acute postprandial study (2 h)	↓ peak postprandial glucose and glucose AUC after both turmeric- and cinnamon-enriched yogurts vs. control	[15]
Turmeric Cinnamon Star anise, Ginger	Polyphenol-rich beverage consumed before standardized bread breakfast	220 mL polyphenol-rich beverage prepared from a single spice (turmeric, cinnamon, star anise or ginger) consumed before a standardized breakfast providing 50 g available carbohydrate; matched control beverage.	Randomized, single-blind crossover; healthy adults (n = 18); acute postprandial study	Turmeric beverage: ↓ early postprandial glycaemia, ↑ early PYY response, ↓ desire to eat and prospective consumption; cinnamon beverage: ↓ early postprandial glycaemia and ↓ prospective consumption; ginger beverage: ↔ glucose, insulin and appetite responses	[191]
**Mixed-Spice Interventions**					
**Spice/Bioactive Compound**	**Food Matrix/** **Dietary Intervention**	**Dose and Mode** **of Administration**	**Study Design**	**Main Findings**	**Ref.**
Turmeric, black pepper	Isoenergetic standardized breakfast	Standardized breakfast containing 1 g turmeric, 1 g black pepper, or a combination of 1 g turmeric + 1 g black pepper; powders incorporated into the meal.	Randomized crossover; healthy adults (n = 20); acute postprandial study (3 h)	Black pepper and black pepper + turmeric: ↓ postprandial glucose (30 min), ↓ hunger and prospective eating ability, ↑ satiety; ↔ gastrointestinal well-being	[192]
Mixed spices: Basil, garlic, fenugreek, ginger, cinnamon, oat and Jerusalem artichoke (TopiGluk^®^ herbal/plant blend)	Functional bread (TopiGluk^®^)	50 g functional bread containing TopiGluk^®^ herbal/spice blend compared with 50 g rye bread	Prospective controlled study; adults with T2DM (n = 97) and healthy controls (n = 16); acute postprandial study (2 h)	↓ postprandial glucose and insulin in T2DM; ↑ subjective satiety; ↔ overall glycaemic response in healthy controls	[193]
Italian herb mix (rosemary, basil, thyme, oregano, parsley), or cinnamon, or pumpkin pie spice (cinnamon, ginger, nutmeg and allspice)	High-fat, high-carbohydrate standardized test meal	Approximately 1 g herbs/spices per 135 kcal of an individually adjusted high-fat, high-carbohydrate Taiwanese pancake meal (≈6 g for the mean 810 kcal meal); participants consumed test meals containing either Italian herb mix, or cinnamon, or pumpkin pie spice, or salt/pepper only (control).	Randomized, single-blind, four-condition crossover; adults with overweight or obesity (n = 25); acute postprandial study (24 h)	Cinnamon: ↓ postprandial glucose and ↓ early insulin response; pumpkin pie spice: ↓ early insulin response and ↑ FMD at 24 h;	[16]
Mixed spices: Turmeric, garlic, ginger, coriander, clove, cumin, red pepper	Curry with rice	180 g curry containing 14.5 g mixed spices served with 200 g cooked rice; approximately 500 kcal; matched spice-free control meal	Randomized, controlled crossover; healthy men (n = 14); acute postprandial study	↑ flow-mediated dilation (FMD); ↔ measured biochemical markers	[194]
Mixed spices: Paprika, oregano, garlic powder, ginger, black pepper, cinnamon, cloves, rosemary	250 g hamburger patty	11.25 g antioxidant spice blend incorporated into a 250 g hamburger patty before cooking	Randomized, controlled crossover; healthy adults (n = 11); acute postprandial study	↓ MDA formation in cooked meat (−71%); altered postprandial plasma MDA profile; ↓ urinary MDA excretion (−49%)	[195]
Mixed spices: Paprika, oregano, garlic powder, ginger, black pepper, cinnamon, cloves, rosemary	250 g hamburger patty	11.25 g antioxidant spice blend incorporated into a 250 g hamburger patty before cooking	Randomized crossover; men with T2DM (n = 18 completed); acute postprandial study	↑ Endothelial function (reactive hyperemia peripheral arterial tonometry); ↓ urinary MDA; ↔ postprandial glucose, insulin and triglycerides	[196]
**Polyspice Research Program**					
**Spice/Bioactive Compound**	**Food Matrix/** **Dietary Intervention**	**Dose and Mode** **of Administration**	**Study Design**	**Main Findings**	**Ref.**
Mixed spices: Turmeric, coriander, cumin, Indian gooseberry (amla), cayenne pepper, cinnamon, clove	Curry with vegetables and rice	0 g, 6 g or 12 g dried seven-spice blend incorporated into curry with vegetables and served with white rice.	Randomized, controlled, three-condition dose–response crossover; healthy men (n = 20; n = 17 for the 6 g condition); acute postprandial study	↑ 3 h total GLP-1 AUC in a dose-dependent manner	[197]
Mixed spices: Turmeric, coriander, cumin, Indian gooseberry (amla), cayenne pepper, cinnamon, clove	Curry with vegetables and rice	0 g, 6 g or 12 g dried seven-spice blend incorporated into curry with vegetables and served with white rice.	Randomized, controlled, three-condition dose–response crossover; healthy men (n = 20; n = 17 for the 6 g condition); acute postprandial study	↓ hunger and desire to eat after both 6 g and 12 g curry meals vs. control; ↔ plasma ghrelin, fullness and prospective eating	[198]
Mixed spices: Turmeric, coriander, cumin, Indian gooseberry (amla), cayenne pepper, cinnamon, clove	Curry with vegetables and rice	0 g, 6 g or 12 g dried seven-spice blend incorporated into curry with vegetables and served with white rice.	Randomized, controlled, three-condition dose–response crossover; healthy men (n = 20); acute postprandial study	↓ 3 h postprandial glucose iAUC and ↑ postprandial triglycerides in a dose-dependent manner	[199]
Mixed spices: Turmeric, coriander, cumin, Indian gooseberry (amla), cayenne pepper, cinnamon, clove	Curry with vegetables and rice	0 g, 6 g or 12 g dried seven-spice blend incorporated into curry with vegetables and served with white rice.	Randomized, controlled, dose–response crossover; healthy men (n = 15); acute study with faecal sampling at baseline and on Day 2	↓ Bacteroides after 6 g and 12 g curry meals vs. control; ↑ Bifidobacterium after the 12 g meal vs. control; ↔ α-diversity	[200]
**Penn State Spice Meal Studies**					
**Spice/Bioactive Compound**	**Food Matrix/** **Dietary Intervention**	**Dose and Mode** **of Administration**	**Study Design**	**Main Findings**	**Ref.**
Mixed spices: Turmeric, paprika, oregano, garlic powder, ginger, black pepper, cinnamon, cloves and rosemary	High-fat, high-carbohydrate meal	14 g dried spice blend incorporated into a 1200 kcal high-fat, high-carbohydrate meal; matched spice-free control meal.	Randomized, controlled, two-period crossover; healthy overweight men (n = 6); 3.5 h postprandial study	↓ postprandial insulin AUC (−21%) and triglyceride AUC (−31%); ↑ FRAP and hydrophilic ORAC; ↔ glucose, total ORAC, lipophilic ORAC and total thiols	[201]
Mixed spices: Turmeric, paprika, oregano, garlic powder, ginger, black pepper, cinnamon, cloves, rosemary	High-fat meal	14.5 g dried spice blend incorporated into a high-fat meal; meal consumed under resting and acute psychological stress conditions.	Randomized crossover; healthy adults (n = 20, including 6 women); acute postprandial study	↓ postprandial triglycerides under resting conditions; effect abolished by acute psychological stress;	[202]
Mixed spices: turmeric, ginger, cinnamon, oregano, parsley, basil, coriander, cumin, red pepper, rosemary, black pepper, bay leaf, thyme	High-saturated-fat, high-carbohydrate meal	2 g or 6 g dried spice blend incorporated into a high-saturated-fat, high-carbohydrate meal; matched spice-free control meal.	Randomized, controlled, three-period crossover; men with overweight or obesity (n = 12); acute postprandial study	6 g spice meal: ↓ LPS-stimulated PBMC secretion of IL-1β, IL-8 and TNF-α; ↔ plasma cytokine concentrations	[203]
Mixed spices: turmeric, ginger, cinnamon, oregano, parsley, basil, coriander, cumin, red pepper, rosemary, black pepper, bay leaf, thyme	High-saturated-fat, high-carbohydrate meal	2 g or 6 g dried spice blend incorporated into a high-saturated-fat, high-carbohydrate meal; matched spice-free control meal.	Randomized, controlled, three-period crossover pilot trial; men with overweight or obesity and increased waist circumference (n = 13); 4 h postprandial study	2 g spice meal: ↓ postprandial triglycerides; 6 g spice meal: ↑ flow-mediated dilation (FMD); ↔ glucose and insulin	[204]

Abbreviations: AUC, area under the concentration–time curve; Cmax, maximum plasma concentration; DMC, demethoxycurcumin; BDMC, bisdemethoxycurcumin; ECBB, encapsulated curcumin plus other polyphenols; ECB, encapsulated curcumin bread; GAE, gallic acid equivalents; T2DM, type 2 diabetes mellitus; iAUC, incremental area under the concentration–time curve; PYY, peptide YY; FMD, flow-mediated dilation; MDA, malondialdehyde; GLP-1, glucagon-like peptide-1; FRAP, ferric reducing antioxidant power; ORAC, oxygen radical absorbance capacity; LPS, lipopolysaccharide; PBMC, peripheral blood mononuclear cells; IL-1β, interleukin-1 beta; IL-8, interleukin-8; TNF-α, tumor necrosis factor alpha; ↑, statistically significant increase or improvement; ↓, statistically significant decrease or reduction; ↔, no statistically significant change. Note: Main findings summarize the principal statistically significant outcomes reported in each study and are not intended to provide a comprehensive description of all measured endpoints.

## Data Availability

No new data were created or analyzed in this study. Data sharing is not applicable to this article.

## References

[1] Rubió L., Motilva M.J., Romero M.P. (2013). Recent Advances in Biologically Active Compounds in Herbs and Spices: A Review of the Most Effective Antioxidant and Anti-Inflammatory Active Principles. Crit. Rev. Food Sci. Nutr..

[2] Sun W., Shahrajabian M.H. (2023). Therapeutic Potential of Phenolic Compounds in Medicinal Plants—Natural Health Products for Human Health. Molecules.

[3] Srinivasan K. (2014). Antioxidant Potential of Spices and Their Active Constituents. Crit. Rev. Food Sci. Nutr..

[4] Alkhatib D.H., Jaleel A., Tariq M.N.M., Feehan J., Apostolopoulos V., Ismail L.C., Stojanovska L., Al Dhaheri A.S. (2022). The Role of Bioactive Compounds from Dietary Spices in the Management of Metabolic Syndrome: An Overview. Nutrients.

[5] Meng W., Chao W., Kaiwei Z., Sijia M., Jiajia S., Shijie X. (2025). Bioactive Compounds from Chinese Herbal Plants for Neurological Health: Mechanisms, Pathways, and Functional Food Applications. Front. Nutr..

[6] Kumar H., Dhalaria R., Guleria S., Sharma R., Cimler R., Dhanjal D.S., Chopra C., Kumar V., Manickam S., Siddiqui S.A. (2025). Advances in the Concept of Functional Foods and Feeds: Applications of Cinnamon and Turmeric as Functional Enrichment Ingredients. Crit. Rev. Food Sci. Nutr..

[7] Khare P., Jagtap S., Jain Y., Baboota R.K., Mangal P., Boparai R.K., Bhutani K.K., Sharma S.S., Premkumar L.S., Kondepudi K.K. (2016). Cinnamaldehyde Supplementation Prevents Fasting-Induced Hyperphagia, Lipid Accumulation, and Inflammation in High-Fat Diet-Fed Mice. BioFactors.

[8] Shahidi F., Pan Y. (2022). Influence of Food Matrix and Food Processing on the Chemical Interaction and Bioaccessibility of Dietary Phytochemicals: A Review. Crit. Rev. Food Sci. Nutr..

[9] Zhang J., Wang H., Ai C., Lu R., Chen L., Xiao J., Teng H. (2024). Food Matrix-Flavonoid Interactions and Their Effect on Bioavailability. Crit. Rev. Food Sci. Nutr..

[10] Vitaglione P., Barone Lumaga R., Ferracane R., Radetsky I., Mennella I., Schettino R., Koder S., Shimoni E., Fogliano V. (2012). Curcumin Bioavailability from Enriched Bread: The Effect of Microencapsulated Ingredients. J. Agric. Food Chem..

[11] Nasef N.A., Loveday S.M., Golding M., Martins R.N., Shah T.M., Clarke M., Coad J., Moughan P.J., Garg M.L., Singh H. (2019). Food Matrix and Co-Presence of Turmeric Compounds Influence Bioavailability of Curcumin in Healthy Humans. Food Funct..

[12] Schönenberger K.A., Ranzini C., Laval J., Bellenger P., Tenon M., Fança-Berthon P. (2025). The Influence of Food Matrices on the Bioavailability of Curcuminoids from a Dried Colloidal Turmeric Suspension: A Randomized, Crossover, Clinical Trial. Food Funct..

[13] Sıçramaz H. (2025). Turmeric-Enriched Yogurt: Increased Antioxidant and Phenolic Contents. Fermentation.

[14] Moreira F.D., Reis C.E.G., Gallassi A.D., Moreira D.C., Welker A.F. (2024). Suppression of the Postprandial Hyperglycemia in Patients with Type 2 Diabetes by a Raw Medicinal Herb Powder Is Weakened When Consumed in Ordinary Hard Gelatin Capsules: A Randomized Crossover Clinical Trial. PLoS ONE.

[15] Pavalakumar D., Jayasinghe M., Edirisinghe M., Wijesekara I., Senadheera S. (2021). *Cinnamomum Zeylanicum* and *Curcuma longa* Incorporated Dairy Yoghurts with Hindered Glycaemic Properties for Healthy People. J. Future Foods.

[16] Huang Y., Tsai M.-F., Thorat R.S., Xiao D., Zhang X., Sandhu A.K., Edirisinghe I., Burton-Freeman B.M. (2022). Endothelial Function and Postprandial Glucose Control in Response to Test-Meals Containing Herbs and Spices in Adults with Overweight/Obesity. Front. Nutr..

[17] Shi L., Xun W., Peng W., Hu H., Cao T., Hou G. (2020). Effect of the Single and Combined Use of Curcumin and Piperine on Growth Performance, Intestinal Barrier Function, and Antioxidant Capacity of Weaned Wuzhishan Piglets. Front. Vet. Sci..

[18] Yashin A., Yashin Y., Xia X., Nemzer B. (2017). Antioxidant Activity of Spices and Their Impact on Human Health: A Review. Antioxidants.

[19] Mackonochie M., Rodriguez-Mateos A., Mills S., Rolfe V. (2023). A Scoping Review of the Clinical Evidence for the Health Benefits of Culinary Doses of Herbs and Spices for the Prevention and Treatment of Metabolic Syndrome. Nutrients.

[20] Liu X.-Y., Nie Y.-K., Liu Y., Chen M. (2025). Cardiovascular Protective Properties of the Natural Product Eugenol. Eur. J. Pharmacol..

[21] Zhang H., Tsao R. (2016). Dietary Polyphenols, Oxidative Stress and Antioxidant and Anti-Inflammatory Effects. Curr. Opin. Food Sci..

[22] Lazaridis D.G., Kitsios A.-P., Koutoulis A.S., Malisova O., Karabagias I.K. (2024). Fruits, Spices and Honey Phenolic Compounds: A Comprehensive Review on Their Origin, Methods of Extraction and Beneficial Health Properties. Antioxidants.

[23] Kiokias S., Oreopoulou V. (2021). A Review of the Health Protective Effects of Phenolic Acids against a Range of Severe Pathologic Conditions (Including Coronavirus-Based Infections). Molecules.

[24] Mirzaei S., Gholami M.H., Zabolian A., Saleki H., Farahani M.V., Hamzehlou S., Far F.B., Sharifzadeh S.O., Samarghandian S., Khan H. (2021). Caffeic Acid and Its Derivatives as Potential Modulators of Oncogenic Molecular Pathways: New Hope in the Fight against Cancer. Pharmacol. Res..

[25] Mu H.N., Zhou Q., Yang R.Y., Tang W.Q., Li H.X., Wang S.M., Li J., Chen W.X., Dong J. (2021). Caffeic Acid Prevents Non-Alcoholic Fatty Liver Disease Induced by a High-Fat Diet through Gut Microbiota Modulation in Mice. Food Res. Int..

[26] Al-Khayri J.M., Sahana G.R., Nagella P., Joseph B.V., Alessa F.M., Al-Mssallem M.Q. (2022). Flavonoids as Potential Anti-Inflammatory Molecules: A Review. Molecules.

[27] Rao P.V., Gan S.H. (2014). Cinnamon: A Multifaceted Medicinal Plant. Evid.-Based Complement. Altern. Med..

[28] Suantawee T., Wesarachanon K., Anantsuphasak K., Daenphetploy T., Thien-Ngern S., Thilavech T., Pasukamonset P., Ngamukote S., Adisakwattana S. (2015). Protein Glycation Inhibitory Activity and Antioxidant Capacity of Clove Extract. J. Food Sci. Technol..

[29] Hewlings S.J., Kalman D.S. (2017). Curcumin: A Review of Its Effects on Human Health. Foods.

[30] Gupta S.C., Prasad S., Kim J.H., Patchva S., Webb L.J., Priyadarsini I.K., Aggarwal B.B. (2011). Multitargeting by Curcumin as Revealed by Molecular Interaction Studies. Nat. Prod. Rep..

[31] Ninkuu V., Aluko O.O., Yan J., Zeng H., Liu G., Zhao J., Li H., Chen S., Dakora F.D. (2025). Phenylpropanoids Metabolism: Recent Insight into Stress Tolerance and Plant Development Cues. Front. Plant Sci..

[32] Liu G., Zhang E., Xiang X., Zhu Y., Tao H., Gu W. (2025). Research Progress on the Structure and Activity of Phenylpropanoid Glycosides. Eur. J. Med. Chem..

[33] Sharma U.K., Sharma A.K., Pandey A.K. (2016). Medicinal Attributes of Major Phenylpropanoids Present in Cinnamon. BMC Complement. Altern. Med..

[34] Adisakwattana S. (2017). Cinnamic Acid and Its Derivatives: Mechanisms for Prevention and Management of Diabetes and Its Complications. Nutrients.

[35] Semwal R.B., Semwal D.K., Combrinck S., Viljoen A.M. (2015). Gingerols and Shogaols: Important Nutraceutical Principles from Ginger. Phytochemistry.

[36] Ballester P., Cerdá B., Arcusa R., Marhuenda J., Yamedjeu K., Zafrilla P. (2022). Effect of Ginger on Inflammatory Diseases. Molecules.

[37] Shahidi F., Athiyappan K.D. (2025). Polyphenol-Polysaccharide Interactions: Molecular Mechanisms and Potential Applications in Food Systems—A Comprehensive Review. Food Prod. Process. Nutr..

[38] Buchweitz M., Speth M., Kammerer D.R., Carle R. (2013). Stabilisation of Strawberry (*Fragaria x ananassa* Duch.) Anthocyanins by Different Pectins. Food Chem..

[39] Liu X., Le Bourvellec C., Renard C.M.G.C. (2020). Interactions between Cell Wall Polysaccharides and Polyphenols: Effect of Molecular Internal Structure. Compr. Rev. Food Sci. Food Saf..

[40] Buchweitz M., Speth M., Kammerer D.R., Carle R. (2013). Impact of Pectin Type on the Storage Stability of Black Currant (*Ribes nigrum* L.) Anthocyanins in Pectic Model Solutions. Food Chem..

[41] Callies O., Hernández Daranas A. (2016). Application of Isothermal Titration Calorimetry as a Tool to Study Natural Product Interactions. Nat. Prod. Rep..

[42] Ye J.H., Jin J., Liang H.L., Lu J.L., Du Y.Y., Zheng X.Q., Liang Y.R. (2009). Using Tea Stalk Lignocellulose as an Adsorbent for Separating Decaffeinated Tea Catechins. Bioresour. Technol..

[43] Le Bourvellec C., Guyot S., Renard C.M.G.C. (2009). Interactions between Apple (*Malus x domestica* Borkh.) Polyphenols and Cell Walls Modulate the Extractability of Polysaccharides. Carbohydr. Polym..

[44] Koh J., Xu Z., Wicker L. (2020). Binding Kinetics of Blueberry Pectin-Anthocyanins and Stabilization by Non-Covalent Interactions. Food Hydrocoll..

[45] Le Bourvellec C., Bouchet B., Renard C.M.G.C. (2005). Non-Covalent Interaction between Procyanidins and Apple Cell Wall Material. Part III: Study on Model Polysaccharides. Biochim. Biophys. Acta Gen. Subj..

[46] Ai C., Zhao C., Xiang C., Zheng Y., Zhong S., Teng H., Chen L. (2023). Gum Arabic as a Sole Wall Material for Constructing Nanoparticle to Enhance the Stability and Bioavailability of Curcumin. Food Chem. X.

[47] Le Bourvellec C., Watrelot A.A., Ginies C., Imberty A., Renard C.M.G.C. (2012). Impact of Processing on the Noncovalent Interactions between Procyanidin and Apple Cell Wall. J. Agric. Food Chem..

[48] Li M., Ndiaye C., Corbin S., Foegeding E.A., Ferruzzi M.G. (2020). Starch-Phenolic Complexes Are Built on Physical CH-π Interactions and Can Persist after Hydrothermal Treatments Altering Hydrodynamic Radius and Digestibility of Model Starch-Based Foods. Food Chem..

[49] Watrelot A.A., Schulz D.L., Kennedy J.A. (2017). Wine Polysaccharides Influence Tannin-Protein Interactions. Food Hydrocoll..

[50] Sun L., Sun J., Liu D., Fu M., Yang X., Guo Y. (2018). The Preservative Effects of Chitosan Film Incorporated with Thinned Young Apple Polyphenols on the Quality of Grass Carp (*Ctenopharyngodon idellus*) Fillets during Cold Storage: Correlation between the Preservative Effects and the Active Properties of the Film. Food Packag. Shelf Life.

[51] Bindon K.A., Bacic A., Kennedy J.A. (2012). Tissue-Specific and Developmental Modifications of Grape Cell Walls Influence the Adsorption of Proanthocyanidins. J. Agric. Food Chem..

[52] Ke Y., Deng L., Dai T., Xiao M., Chen M., Liang R., Liu W., Liu C., Chen J. (2022). Effects of Cell Wall Polysaccharides on the Bioaccessibility of Carotenoids, Polyphenols, and Minerals: An Overview. Crit. Rev. Food Sci. Nutr..

[53] Liu Q., Zou X., Yi Y., Sun Y., Wang H., Jiang X., Peng K. (2023). Physicochemical and Functional Changes in Lotus Root Polysaccharide Associated with Noncovalent Binding of Polyphenols. Foods.

[54] Fernandes A., Mateus N., de Freitas V. (2023). Polyphenol-Dietary Fiber Conjugates from Fruits and Vegetables: Nature and Biological Fate in a Food and Nutrition Perspective. Foods.

[55] Liang J., Li H., Han M., Gao Z. (2025). Polysaccharide-Polyphenol Interactions: A Comprehensive Review from Food Processing to Digestion and Metabolism. Crit. Rev. Food Sci. Nutr..

[56] Quan T.H., Benjakul S., Sae-leaw T., Balange A.K., Maqsood S. (2019). Protein–Polyphenol Conjugates: Antioxidant Property, Functionalities and Their Applications. Trends Food Sci. Technol..

[57] Huynh H.D., Thi T.H.T., Thi T.X.T., Nargotra P., Wang H.M.D., Liu Y.C., Kuo C.H. (2026). Recent Insights into Protein-Polyphenol Complexes: Molecular Mechanisms, Processing Technologies, Synergistic Bioactivities, and Food Applications. Molecules.

[58] Li N., Shou Z., Yang S., Cheng X., Chen C., Zheng S., Shi Y., Tang H. (2023). Subtle Distinction in Molecular Structure of Flavonoids Leads to Vastly Different Coating Efficiency and Mechanism of Metal-Polyphenol Networks with Excellent Antioxidant Activities. Colloids Surf. B Biointerfaces.

[59] Liu J., Zhang Y., Liu J., Zhang H., Gong L., Li Z., Liu H., Wang Z. (2024). Effect of Non-Covalently Bound Polyphenols on the Structural and Functional Properties of Wheat Germ Protein. Food Hydrocoll..

[60] Chen H.H., Jiang H., Chen Y., Qu Y.P., Wang Y.S. (2022). A PH-Controlled Curcumin-Loaded Emulsion Stabilized by Pea Protein Isolate-Maltodextrin-Epigallocatechin-3-Gallate: Physicochemical Properties and in Vitro Release Properties. Colloids Surf. A Physicochem. Eng. Asp..

[61] Bayati M., Poojary M.M. (2025). Polyphenol Autoxidation and Prooxidative Activity Induce Protein Oxidation and Protein-Polyphenol Adduct Formation in Model Systems. Food Chem..

[62] Li K., Yuan X., Zhao J., Ren J., Ma L., Liao X., Hu X., Chen F., Ji J. (2024). Covalent Conjugate of Pea Protein Induced by Cyanidin-3-O-Glucoside Quinone: The Structural Formation and Functional Properties. Food Hydrocoll..

[63] Ai M., Zhou Q., Guo S., Ling Z., Zhou L., Fan H., Cao Y., Jiang A. (2019). Effects of Tea Polyphenol and Ca(OH)2 on the Intermolecular Forces and Mechanical, Rheological, and Microstructural Characteristics of Duck Egg White Gel. Food Hydrocoll..

[64] Yilmaz H., Gultekin Subasi B., Celebioglu H.U., Ozdal T., Capanoglu E. (2022). Chemistry of Protein-Phenolic Interactions Toward the Microbiota and Microbial Infections. Front. Nutr..

[65] Wang Q., Tang Y., Yang Y., Lei L., Lei X., Zhao J., Zhang Y., Li L., Wang Q., Ming J. (2022). The Interaction Mechanisms, and Structural Changes of the Interaction between Zein and Ferulic Acid under Different PH Conditions. Food Hydrocoll..

[66] Zhang K., Zhu K., Jiang R., Huang J., Wang D., Ho C.-T., Wan X., Wang Y. (2025). Discovery and Identification of the Temperature Dependent EGCG and β-Lactoglobulin Covalent Conjugate in Green Tea and Milk Mixture. Food Hydrocoll..

[67] Ji F., Liu H., Wang C., Guo N., Shen Y., Luo S., Jiang S., Zheng Z. (2024). Remodeling the Structure of Soy Protein Fibrils to Hydrogels for Co-Encapsulation of (−)-Epigallocatechin Gallate (EGCG) and Curcumin: Role of EGCG. Food Hydrocoll..

[68] Dai S., Liao P., Wang Y., Tian T., Tong X., Lyu B., Cheng L., Miao L., Qi W., Jiang L. (2023). Soy Protein Isolate-Catechin Non-Covalent and Covalent Complexes: Focus on Structure, Aggregation, Stability and in Vitro Digestion Characteristics. Food Hydrocoll..

[69] Sun H., Liao F., Tian Y., Lei Y., Fu Y., Wang J. (2024). Molecular-Scale Investigations Reveal the Effect of Natural Polyphenols on BAX/Bcl-2 Interactions. Int. J. Mol. Sci..

[70] Wang N., Ma Z., Ma L., Zhang Y., Zhang K., Ban Q., Wang X. (2023). Synergistic Modification of Structural and Functional Characteristics of Whey Protein Isolate by Soybean Isoflavones Non-Covalent Binding and Succinylation Treatment: A Focus on Emulsion Stability. Food Hydrocoll..

[71] Yin B., Wu Q., Zheng Z., Wang R., Zhao Y., Zhao W., Wang D., Qin P., Zhao S., Kan J. (2025). Effects of Non-Covalent Binding of Different Proteins and Apple Polyphenols on Structure and Functional Properties. Food Hydrocoll..

[72] Kanwal Q., Ahmed M., Hamza M., Ahmad M., Rehman A.U., Yousaf N., Javaid A., Anwar A., Khan I.H., Muddassar M. (2023). Curcumin Nanoparticles: Physicochemical Fabrication, Characterization, Antioxidant, Enzyme Inhibition, Molecular Docking and Simulation Studies. RSC Adv..

[73] Li Y., Li M., Li Q., Geng F., Wang Q., Gan N., Li S., Wu D. (2024). Revealing the Potential of Theaflavin to Reduce Egg Allergenicity at the Molecular Level: A Study of the Binding Affinity of Theaflavin to Egg White Lysozyme. Food Bioprocess Technol..

[74] Martinez-Gonzalez A.I., Díaz-Sánchez Á.G., De La Rosa L.A., Vargas-Requena C.L., Bustos-Jaimes I., Alvarez-Parrilla E. (2017). Polyphenolic Compounds and Digestive Enzymes: In Vitro Non-Covalent Interactions. Molecules.

[75] Ozdal T., Yalcinkaya İ.E., Toydemir G., Capanoglu E. (2018). Polyphenol-Protein Interactions and Changes in Functional Properties and Digestibility. Encyclopedia of Food Chemistry.

[76] Siebert K.J. (2009). Haze in Beverages. Adv. Food Nutr. Res..

[77] Karonen M. (2022). Insights into Polyphenol–Lipid Interactions: Chemical Methods, Molecular Aspects and Their Effects on Membrane Structures. Plants.

[78] Nie R.-Z., Dang M.-Z., Ge Z.-Z., Huo Y.-Q., Yu B., Tang S.-W. (2021). Influence of the Gallate Moiety on the Interactions between Green Tea Polyphenols and Lipid Membranes Elucidated by Molecular Dynamics Simulations. Biophys. Chem..

[79] Pruchnik H., Bonarska-Kujawa D., Żyłka R., Oszmiański J., Kleszczyńska H. (2018). Application of the DSC and Spectroscopy Methods in the Analysis of the Protective Effect of Extracts from the Blueberry Fruit of the Genus Vaccinium in Relation to the Lipid Membrane. J. Therm. Anal. Calorim..

[80] Scheidt H.A., Pampel A., Nissler L., Gebhardt R., Huster D. (2004). Investigation of the Membrane Localization and Distribution of Flavonoids by High-Resolution Magic Angle Spinning NMR Spectroscopy. Biochim. Biophys. Acta Biomembr..

[81] Rothwell J.A., Day A.J., Morgan M.R.A. (2005). Experimental Determination of Octanol-Water Partition Coefficients of Quercetin and Related Flavonoids. J. Agric. Food Chem..

[82] Cao H., Jing X., Wu D., Shi Y. (2013). Methylation of Genistein and Kaempferol Improves Their Affinities for Proteins. Int. J. Food Sci. Nutr..

[83] Sanver D., Murray B.S., Sadeghpour A., Rappolt M., Nelson A.L. (2016). Experimental Modeling of Flavonoid-Biomembrane Interactions. Langmuir.

[84] Ulrih N.P., Maričić M., Ota A., Šentjurc M., Abram V. (2015). Kaempferol and Quercetin Interactions with Model Lipid Membranes. Food Res. Int..

[85] Zhu W., Khalifa I., Peng J., Li C. (2018). Position and Orientation of Gallated Proanthocyanidins in Lipid Bilayer Membranes: Influence of Polymerization Degree and Linkage Type. J. Biomol. Struct. Dyn..

[86] Neves A.R., Nunes C., Reis S. (2016). Resveratrol Induces Ordered Domains Formation in Biomembranes: Implication for Its Pleiotropic Action. Biochim. Biophys. Acta Biomembr..

[87] Galiano V., Villalaín J. (2015). Oleuropein Aglycone in Lipid Bilayer Membranes. A Molecular Dynamics Study. Biochim. Biophys. Acta Biomembr..

[88] Phan H.T.T., Yoda T., Chahal B., Morita M., Takagi M., Vestergaard M.C. (2014). Structure-Dependent Interactions of Polyphenols with a Biomimetic Membrane System. Biochim. Biophys. Acta Biomembr..

[89] Reis A., Perez-Gregorio R., Mateus N., de Freitas V. (2020). Interactions of Dietary Polyphenols with Epithelial Lipids: Advances from Membrane and Cell Models in the Study of Polyphenol Absorption, Transport and Delivery to the Epithelium. Crit. Rev. Food Sci. Nutr..

[90] Cianciosi D., Forbes-Hernández T.Y., Regolo L., Alvarez-Suarez J.M., Navarro-Hortal M.D., Xiao J., Quiles J.L., Battino M., Giampieri F. (2022). The Reciprocal Interaction between Polyphenols and Other Dietary Compounds: Impact on Bioavailability, Antioxidant Capacity and Other Physico-Chemical and Nutritional Parameters. Food Chem..

[91] Kajiya K., Kumazawa S., Nakayama T. (2002). Effects of External Factors on the Interaction of Tea Catechins with Lipid Bilayers. Biosci. Biotechnol. Biochem..

[92] Lamothe S., Langlois A., Bazinet L., Couillard C., Britten M. (2016). Antioxidant Activity and Nutrient Release from Polyphenol-Enriched Cheese in a Simulated Gastrointestinal Environment. Food Funct..

[93] Kim E., Ban C., Kim S.O., Lim S., Choi Y.J. (2022). Applications and Perspectives of Polyphenol-Loaded Solid Lipid Nanoparticles and Nanostructured Lipid Carriers for Foods. Food Sci. Biotechnol..

[94] Vazhappilly C.G., Amararathna M., Cyril A.C., Linger R., Matar R., Merheb M., Ramadan W.S., Radhakrishnan R., Rupasinghe H.P.V. (2021). Current Methodologies to Refine Bioavailability, Delivery, and Therapeutic Efficacy of Plant Flavonoids in Cancer Treatment. J. Nutr. Biochem..

[95] Tan O.J., Loo H.L., Thiagarajah G., Palanisamy U.D., Sundralingam U. (2021). Improving Oral Bioavailability of Medicinal Herbal Compounds through Lipid-Based Formulations—A Scoping Review. Phytomedicine.

[96] Mandić L., Sadžak A., Strasser V., Baranović G., Jurašin D.D., Sikirić M.D., Šegota S. (2019). Enhanced Protection of Biological Membranes during Lipid Peroxidation: Study of the Interactions between Flavonoid Loaded Mesoporous Silica Nanoparticles and Model Cell Membranes. Int. J. Mol. Sci..

[97] Tsagkaris A.S., Louckova A., Jaegerova T., Tokarova V., Hajslova J. (2022). The In Vitro Inhibitory Effect of Selected Asteraceae Plants on Pancreatic Lipase Followed by Phenolic Content Identification through Liquid Chromatography High Resolution Mass Spectrometry (LC-HRMS). Int. J. Mol. Sci..

[98] Anselmo S., Bonaccorso E., Gangemi C., Sancataldo G., Conti Nibali V., D’Angelo G. (2025). Lipid Rafts in Signalling, Diseases, and Infections: What Can Be Learned from Fluorescence Techniques?. Membranes.

[99] Choe E. (2020). Roles and Action Mechanisms of Herbs Added to the Emulsion on Its Lipid Oxidation. Food Sci. Biotechnol..

[100] Zagórska J., Pietrzak K., Kukula-Koch W., Czop M., Laszuk J., Koch W. (2023). Influence of Diet on the Bioavailability of Active Components from *Zingiber Officinale* Using an In Vitro Digestion Model. Foods.

[101] Zubaidah E., Nisak Y.K., Wijayanti S.A., Christianty R.A. (2021). Characteristic of Microbiological, Chemical, and Antibacterial Activity of Turmeric (*Curcuma longa*) Kombucha. IOP Conf. Ser. Earth Environ. Sci..

[102] Rak K., Kolniak-Ostek J., Gajda R., Marcinkiewicz K., Nemś A., Raczkowska E. (2025). Antioxidant Activity, Total Polyphenol Content, and Mineral Composition of Milk Beverages Fortified with Spice Mixtures (Clove, Cinnamon, and Turmeric) and Natural Sweeteners (Erythritol and Stevia): Evidence of Synergistic or Antagonistic Effects of Compounds. Int. J. Mol. Sci..

[103] Karigidi K.O., Akintimehin E.S., Akinyemi O., Fapetu A.P., Adetuyi F.O. (2022). Nutritional, Antioxidant, Antidiabetic, and Oxidative Stability Properties of Turmeric (*Curcuma longa*) Supplemented Muffins. J. Food Process. Preserv..

[104] de Castro Cogle K., Kubo M.T.K., Merlier F., Josse A., Anastasiadi M., Mohareb F.R., Rossi C. (2024). Probabilistic Modelling of the Food Matrix Effects on Curcuminoid’s In Vitro Oral Bioaccessibility. Foods.

[105] Bouatenin K.M.J.P., Camara F., Tohoyessou Y.M.G., Hermann Coulibaly W., Boli Z.B.I.A., Ouattara G.A., Koussemon M. (2023). Contribution to the Improvement of the Nutritional and Functional Properties of Bread by Incorporating Cinnamon Powder (*Cinnamomum verum*). Food Sci. Nutr..

[106] Issaoui M., Nesrine M., Flamini G., Delgado A. (2021). Enrichment of White Flour with Spices Positively Impacts Safety and Consumer Acceptance of Bread. Int. J. Food Sci. Technol..

[107] Bordbar Lomer B., Ghannadiasl F. (2025). Enrichment of Oil Cake with Cinnamon Extract Positively Effects Antioxidant Activity and Textural Profile. Food Sci. Nutr..

[108] Aghvami M., Mohammadi A., Khaniki G.J., Ahmadi M., Moazzen M., Arabameri M., Shariatifar N. (2023). Investigation of Cocoa and Cinnamon Effect on Acrylamide Formation in Cakes Production Using GC/MS Method: A Risk Assessment Study. Food Chem. X.

[109] Tang P.L., Chen Y.T., Qin J., Hou X., Deng J. (2020). Effect of Cinnamon Bark and Twig Extracts on the Chemical, Physicochemical and Antioxidant Properties of Fermented Milk. J. Food Meas. Charact..

[110] Tang P.L., Cham X.Y., Hou X., Deng J. (2022). Potential Use of Waste Cinnamon Leaves in Stirred Yogurt Fortification. Food Biosci..

[111] Gonçalves R.F.S., Fernandes J.M., Martins J.T., Vieira J.M., Abreu C.S., Gomes J.R., Vicente A.A., Pinheiro A.C. (2024). Incorporation of Curcumin-Loaded Solid Lipid Nanoparticles into Yogurt: Tribo-Rheological Properties and Dynamic in Vitro Digestion. Food Res. Int..

[112] Abdullah R., Arshad H., Kaleem A., Iqtedar M., Aftab M., Saleem F. (2023). Assessment of Angiotensin Converting Enzyme Inhibitory Activity and Quality Attributes of Yoghurt Enriched with *Cinnamomum verum*, *Elettaria cardamomum*, *Beta vulgaris* and *Brassica oleracea*. Saudi J. Biol. Sci..

[113] Wihansah R.R.S., Pazra D.F., Wahyuningsih, Handayani K.S. (2022). Assesment of the Antidiabetic Activity and Characteristics of Cow’s Milk Yogurt Enhanced with Herbs Extracts. IOP Conf. Ser. Earth Environ. Sci..

[114] Güldane M. (2025). Effect of Enrichment with Spice Extracts on Yoghurt Quality: Optimizing the Extraction Process and Sensory Properties: Yoghurt Enriched with Spice Extracts. J. Food Meas. Charact..

[115] Oguntoyinbo O.O., Babarinsa O.A., Otesile I.I. (2025). Production and Evaluation of Yoghurt Produced from Tiger Nut Enriched with Cinnamon. Bull. Univ. Agric. Sci. Vet. Med. Cluj-Napoca. Food Sci. Technol..

[116] Indiarto R., Situmorang A.K.N., Harunaningtyas A., Arifin H.R., Subroto E., Herawati E.R.N., Djali M., Mahani, Muhammad D.R.A. (2024). Reformulation of White Chocolate with Soy- and Coconut-Based Vegetable Ingredients Incorporating Encapsulated Cinnamon Extract: Investigation of Physicochemical, Antioxidant, and Sensory Properties. Int. J. Food Prop..

[117] Helal A., Tagliazucchi D., Verzelloni E., Conte A. (2014). Bioaccessibility of Polyphenols and Cinnamaldehyde in Cinnamon Beverages Subjected to in Vitro Gastro-Pancreatic Digestion. J. Funct. Foods.

[118] Indarto I., Astuti S.D., Rudini M., Pambudi W. (2020). Increasing Antioxidant Activity and Organoleptic Properties of Soursop Leaf Tea (*Annona muricata* Linn.) by Adding Cinnamon Powder (*Cinnamomum burmanni*). Biosf. J. Tadris Biol..

[119] Amjad A., Sohaib M., Nawaz H., Javed M.S., Shah M., Shah F.U.H., Tariq M.R., Sajid M.W., Khan A.A., Bilal M. (2022). Assessment of Rheological and Quality Characteristics of Bread Made by the Addition of Ginger Powder in Wheat Flour. Food Sci. Technol..

[120] Marak N.R., Malemnganbi C.C., Marak C.R., Mishra L.K. (2019). Functional and Antioxidant Properties of Cookies Incorporated with Foxtail Millet and Ginger Powder. J. Food Sci. Technol..

[121] Kaushal M., Vaidya D., Gupta A., Kaushik R., Verma A.K. (2019). Bioactive Compounds and Acceptance of Cookies Supplemented with Ginger Flour. J. Pharmacogn. Phytochem..

[122] Yang H., Li L., Yin Y., Li B., Zhang X., Jiao W., Liang Y. (2019). Effect of Ground Ginger on Dough and Biscuit Characteristics and Acrylamide Content. Food Sci. Biotechnol..

[123] Felfoul I., Borchani M., Samet-Bali O., Attia H., Ayadi M.A. (2017). Effect of Ginger (*Zingiber officinalis*) Addition on Fermented Bovine Milk: Rheological Properties, Sensory Attributes and Antioxidant Potential. J. New Sci. Agric. Biotechnol..

[124] Gabbi D.K., Bajwa U., Goraya R.K. (2018). Physicochemical, Melting and Sensory Properties of Ice Cream Incorporating Processed Ginger (*Zingiber officinale*). Int. J. Dairy Technol..

[125] Kuswardhani N., Mukti N.P., Sari P. (2021). Antioxidant and Sensory Properties of Ready to Drink Coffee-Ginger Made from Decaffeinated and Non-Decaffeinated Robusta Coffee Beans. IOP Conf. Ser. Earth Environ. Sci..

[126] Mancini S., Paci G., Fratini F., Torracca B., Nuvoloni R., Dal Bosco A., Roscini V., Preziuso G. (2017). Improving Pork Burgers Quality Using *Zingiber Officinale Roscoe* Powder (Ginger). Meat Sci..

[127] Mancini S., Preziuso G., Dal Bosco A., Roscini V., Parisi G., Paci G. (2017). Modifications of Fatty Acids Profile, Lipid Peroxidation and Antioxidant Capacity in Raw and Cooked Rabbit Burgers Added with Ginger. Meat Sci..

[128] Partio E.K.U., Tedjakusuma F., Surya R. (2023). Analysis of Antioxidant and Hedonic Acceptance of Turmeric Extract-Enriched Milk. IOP Conf. Ser. Earth Environ. Sci..

[129] Qazi H.J., Ye A., Acevedo-Fani A., Singh H. (2022). Impact of Recombined Milk Systems on Gastrointestinal Fate of Curcumin Nanoemulsion. Front. Nutr..

[130] Hasneen D.F., Zaki N.L., Abbas M.S., Soliman A.S., Ashoush I.S., Fayed A.E. (2020). Comparative Evaluation of Some Herbs and Their Suitability for Skimmed Milk Yoghurt and Cast Kariesh Cheese Fortification as Functional Foods. Ann. Agric. Sci..

[131] de Britto G.C.S., Bécker G., Soares W.P., Nascimento E., Rodrigues E.C., Picanço N.F.M., de Faria R.A.P.G., Scabora M.H. (2020). Bioactive Compounds and Physicochemical Properties of Dairy Products Supplemented with Plantain and Turmeric. J. Food Process. Preserv..

[132] Maji S., Ray P.R., Ghatak P.K., Chakraborty C. (2018). Total Phenolic Content (TPC) and Quality of Herbal Lassi Fortified with Turmeric (*Curcuma longa*) Extract. Asian J. Dairy Food Res..

[133] Gyebi G., Shaibu R., Fabusiwa M., Olaiya C., Ogunyemi O. (2021). Antioxidant, Nutritional, and Physicochemical Quality of Yoghurt Produced from a Milk-Based Fermentation Mix Enhanced with Food Spices. Croat. J. Food Sci. Technol..

[134] Mbaeyi-Nwaoha I.E., Uzoigwe A.C., Onyeaka H. (2024). Quality Evaluation of Herbal Yoghurt Produced Using Cinnamon (*Cinnamon cassia*), Garlic (*Allium sativum*) and Ginger (*Zingiber officinale*). J. Food Qual..

[135] Aydin E. (2022). Evaluation of Phenolic Acid, Total Phenolic Content, Antioxidant Capacity and in-Vitro Simulated Bioaccessibility of Healthy Snack: Aromatized Pumpkin Chips. Emir. J. Food Agric..

[136] Okur Ö.D. (2023). Utilization of Natural Plant Sources in a Traditional Dairy Dessert, Muhallebi. Cogent Food Agric..

[137] Omoarukhe E.D., Harbourne N., Jauregi P. (2023). The Effect of Cinnamon and Ginger Spices on Anthocyanins in Sweetened Roselle Beverages. Beverages.

[138] Fauziah I.N., Prangdimurti E., Palupi N.S. (2023). Bioaccessibility of Antioxidant Capacity of Wedang Uwuh a Traditional Indonesian Beverage by Gastrointestinal Digestion. Curr. Res. Nutr. Food Sci..

[139] Alegría A., Garcia-Llatas G., Cilla A. (2015). Static Digestion Models: General Introduction. The Impact of Food Bioactives on Health: In Vitro and Ex Vivo Models.

[140] Tolve R., Simonato B. (2024). Fortified Cereal-Based Foodstuffs: Technological, Sensory, and Nutritional Properties. Foods.

[141] Kamali Rousta L., Bodbodak S., Nejatian M., Ghandehari Yazdi A.P., Rafiee Z., Xiao J., Jafari S.M. (2021). Use of Encapsulation Technology to Enrich and Fortify Bakery, Pasta, and Cereal-Based Products. Trends Food Sci. Technol..

[142] Gottardi D., Bukvicki D., Prasad S., Tyagi A.K. (2016). Beneficial Effects of Spices in Food Preservation and Safety. Front. Microbiol..

[143] Agrahar-Murugkar D. (2020). Food to Food Fortification of Breads and Biscuits with Herbs, Spices, Millets and Oilseeds on Bio-Accessibility of Calcium, Iron and Zinc and Impact of Proteins, Fat and Phenolics. LWT.

[144] Esposito F., Velotto S., Rea T., Stasi T., Cirillo T. (2020). Occurrence of Acrylamide in Italian Baked Products and Dietary Exposure Assessment. Molecules.

[145] Zhu Y., Zhao Y., Wang P., Ahmedna M., Sang S. (2015). Bioactive Ginger Constituents Alleviate Protein Glycation by Trapping Methylglyoxal. Chem. Res. Toxicol..

[146] Sulieman A.M.E., Abdallah E.M., Alanazi N.A., Ed-Dra A., Jamal A., Idriss H., Alshammari A.S., Shommo S.A.M. (2023). Spices as Sustainable Food Preservatives: A Comprehensive Review of Their Antimicrobial Potential. Pharmaceuticals.

[147] Martínez-Graciá C., González-Bermúdez C.A., Cabellero-Valcárcel A.M., Santaella-Pascual M., Frontela-Saseta C. (2015). Use of Herbs and Spices for Food Preservation: Advantages and Limitations. Curr. Opin. Food Sci..

[148] Milagres de Almeida J., Crippa B.L., Martins Alencar de Souza V.V., Perez Alonso V.P., da Motta Santos Júnior E., Siqueira Franco Picone C., Prata A.S., Cirone Silva N.C. (2023). Antimicrobial Action of Oregano, Thyme, Clove, Cinnamon and Black Pepper Essential Oils Free and Encapsulated against Foodborne Pathogens. Food Control.

[149] Rahmatalla S.A., Alazeem L.A., Abdalla M.O.M. (2017). Microbiological Quality of Set Yoghurt Supplemented with Turmeric Powder (*Curcuma longa*) During Storage. Asian J. Agric. Food Sci..

[150] (2024). Standard for Fermented Milks.

[151] Shori A.B., Baba A.S. (2015). Survival of *Bifidobacterium Bifidum* in Cow- and Camel-Milk Yogurts Enriched with *Cinnamomum verum* and *Allium sativum*. J. Assoc. Arab. Univ. Basic Appl. Sci..

[152] Gunes-Bayir A., Bilgin M.G., Guclu D., Pogda S., Dadak A. (2022). Preparation and Evaluation of Novel Functional Fermented Dairy Products Containing Propolis and Cinnamon. J. Food Sci. Technol..

[153] Ghasemi B., Varidi M.J., Varidi M., Kazemi-Taskooh Z., Emami S.A. (2022). The Effect of Plant Essential Oils on Physicochemical Properties of Chicken Nuggets. J. Food Meas. Charact..

[154] Tajik H., Farhangfar A., Moradi M., Razavi Rohani S.M. (2014). Effectiveness of Clove Essential Oil and Grape Seed Extract Combination on Microbial and Lipid Oxidation Characteristics of Raw Buffalo Patty During Storage at Abuse Refrigeration Temperature. J. Food Process. Preserv..

[155] Abdel-Aziz M.E., Morsy N.F.S. (2015). Keeping Quality of Frozen Beef Patties by Marjoram and Clove Essential Oils. J. Food Process. Preserv..

[156] Sharma H., Mendiratta S.K., Agarwal R.K., Kumar S., Soni A. (2017). Evaluation of Anti-Oxidant and Anti-Microbial Activity of Various Essential Oils in Fresh Chicken Sausages. J. Food Sci. Technol..

[157] Harmankaya S., Harmankaya A., İşbarali K., Paksoy Ö.İ. (2024). Effect of Rosemary and Clove Essential Oils on Lipid Oxidation, Microbial, Sensorial Properties and Storage Stability of Kavurma, a Cooked Meat Product. Czech J. Food Sci..

[158] Silva H.D., Poejo J., Pinheiro A.C., Donsì F., Serra A.T., Duarte C.M.M., Ferrari G., Cerqueira M.A., Vicente A.A. (2018). Evaluating the Behaviour of Curcumin Nanoemulsions and Multilayer Nanoemulsions during Dynamic in Vitro Digestion. J. Funct. Foods.

[159] Li Y., Xu R., Xiu H., Feng J., Jin Park H., Prabhakar H., Kong F. (2022). Effect of Cinnamon on Starch Hydrolysis of Rice Pudding: Comparing Static and Dynamic in Vitro Digestion Models. Food Res. Int..

[160] Cao X., Han Y., Li F., Li Z., McClements D.J., He L., Decker E.A., Xing B., Xiao H. (2019). Impact of Protein-Nanoparticle Interactions on Gastrointestinal Fate of Ingested Nanoparticles: Not Just Simple Protein Corona Effects. NanoImpact.

[161] Mirzaee F., Hosseinzadeh L., Ashrafi-Kooshk M.R., Esmaeili S., Ghobadi S., Farzaei M.H., Zad-Bari M.R., Khodarahmi R. (2018). Diverse Effects of Different “Protein-Based” Vehicles on the Stability and Bioavailability of Curcumin: Spectroscopic Evaluation of the Antioxidant Activity and Cytotoxicity In Vitro. Protein Pept. Lett..

[162] Wang W., Huang Z., Li Y., Wang W., Shi J., Fu F., Huang Y., Pan X., Wu C. (2021). Impact of Particle Size and PH on Protein Corona Formation of Solid Lipid Nanoparticles: A Proof-of-Concept Study. Acta Pharm. Sin. B.

[163] Jin Y., Yu Y., Qi Y., Wang F., Yan J., Zou H. (2016). Peptide Profiling and the Bioactivity Character of Yogurt in the Simulated Gastrointestinal Digestion. J. Proteom..

[164] Engström M.T., Virtanen V., Salminen J.P. (2022). Influence of the Hydrolyzable Tannin Structure on the Characteristics of Insoluble Hydrolyzable Tannin-Protein Complexes. J. Agric. Food Chem..

[165] Flory S., Sus N., Haas K., Jehle S., Kienhöfer E., Waehler R., Adler G., Venturelli S., Frank J. (2021). Increasing Post-Digestive Solubility of Curcumin Is the Most Successful Strategy to Improve Its Oral Bioavailability: A Randomized Cross-Over Trial in Healthy Adults and In Vitro Bioaccessibility Experiments. Mol. Nutr. Food Res..

[166] Liang Y., Matia-Merino L., Patel H., Ye A., Gillies G., Golding M. (2014). Effect of Sugar Type and Concentration on the Heat Coagulation of Oil-in-Water Emulsions Stabilized by Milk-Protein-Concentrate. Food Hydrocoll..

[167] De Freitas V., Carvalho E., Mateus N. (2003). Study of Carbohydrate Influence on Protein-Tannin Aggregation by Nephelometry. Food Chem..

[168] Hagerman A.E., Butler L.G. (1980). Determination of Protein in Tannin-Protein Precipitates. J. Agric. Food Chem..

[169] Sharma P., Verma P.K., Sood S., Yousuf R., Kumar A., Raina R., Shabbir M.A., Bhat Z.F. (2023). Protective Effect of Quercetin and Ginger (*Zingiber officinale*) Extract against Dimethoate Potentiated Fluoride-Induced Nephrotoxicity in Rats. Foods.

[170] Akimoto M., Iizuka M., Kanematsu R., Yoshida M., Takenaga K. (2015). Anticancer Effect of Ginger Extract against Pancreatic Cancer Cells Mainly through Reactive Oxygen Species-Mediated Autotic Cell Death. PLoS ONE.

[171] Ahmed S.S., Kumar M.R. (2023). Elucidation of Antidiabetic Mechanism of *Centella Asiatica* and *Zingiber Officinale*-An In-Vitro and In-Vivo Approach. J. Res. Pharm..

[172] Gutierres V.O., Pinheiro C.M., Assis R.P., Vendramini R.C., Pepato M.T., Brunetti I.L. (2012). Curcumin-Supplemented Yoghurt Improves Physiological and Biochemical Markers of Experimental Diabetes. Br. J. Nutr..

[173] Koh Y.C., Hsu H.W., Ho P.Y., Hsu K.Y., Lin W.S., Nagabhushanam K., Ho C.T., Pan M.H. (2024). Structural Variances in Curcumin Degradants: Impact on Obesity in Mice. J. Agric. Food Chem..

[174] Banji D., Banji O.J.F., Srinivas K. (2021). Neuroprotective Effect of Turmeric Extract in Combination with Its Essential Oil and Enhanced Brain Bioavailability in an Animal Model. BioMed Res. Int..

[175] Gunawan S., Soetikno V., Purwaningsih E.H., Ferdinal F., Wuyung P.E., Ramadhani D. (2024). 6-Gingerol, a Bioactive Compound of *Zingiber Officinale*, Ameliorates High-Fat High-Fructose Diet-Induced Non-Alcoholic Related Fatty Liver Disease in Rats. J. Exp. Pharmacol..

[176] Okamoto M., Irii H., Tahara Y., Ishii H., Hirao A., Udagawa H., Hiramoto M., Yasuda K., Takanishi A., Shibata S. (2011). Synthesis of a New [6]-Gingerol Analogue and Its Protective Effect with Respect to the Development of Metabolic Syndrome in Mice Fed a High-Fat Diet. J. Med. Chem..

[177] Cheng D.M., Kuhn P., Poulev A., Rojo L.E., Lila M.A., Raskin I. (2012). In Vivo and in Vitro Antidiabetic Effects of Aqueous Cinnamon Extract and Cinnamon Polyphenol-Enhanced Food Matrix. Food Chem..

[178] Pérez M.F.R., Lira A.Q., Martini J.P., Martínez M.A., Simental S.S., Rodríguez R.H.A., López J.O., Cortés R.C., Munguía A.R. (2024). Physicochemical Characterization of Yogurt Fortified with Microencapsulated Cinnamon (*Cinnamomum zeylanicum*) and Its Effects on Metabolic Syndrome Induced in Rabbits (*Oryctolagus cuniculus*). J. Med. Food.

[179] Hameed A., Ishtiaq F., Zeeshan M., Akhtar S., Ismail T., Ahmad R.S., Amir M., Anwar M.J. (2023). Combined Antidiabetic Potential of Camel Milk Yogurt with *Cinnamomum verum* and Stevia Rebaudiana by Using Rodent Modelling. J. Food Sci. Technol..

[180] Zuo J., Zhao D., Yu N., Fang X., Mu Q., Ma Y., Mo F., Wu R., Ma R., Wang L. (2017). Cinnamaldehyde Ameliorates Diet-Induced Obesity in Mice by Inducing Browning of White Adipose Tissue. Cell. Physiol. Biochem..

[181] Aly A.A., Zaky E.A., Mahmoud H.A., Alrefaei A.F., Hameed A.M., Alessa H., Alsimaree A.A., Aljohani M., El-Bahy S.M., Kadasah S. (2021). The Impact of Addition Oats (*Avena sativa*) and Cinnamon on Cookies and Their Biological Effects on Rats Treated with Cirrhosis by CCL4. Saudi J. Biol. Sci..

[182] Tamura Y., Iwasaki Y., Narukawa M., Watanabe T. (2012). Ingestion of Cinnamaldehyde, a TRPA1 Agonist, Reduces Visceral Fats in Mice Fed a High-Fat and High-Sucrose Diet. J. Nutr. Sci. Vitaminol..

[183] Li M., Zhao Y., Wang Y., Geng R., Fang J., Kang S.G., Huang K., Tong T. (2022). Eugenol, A Major Component of Clove Oil, Attenuates Adiposity, and Modulates Gut Microbiota in High-Fat Diet-Fed Mice. Mol. Nutr. Food Res..

[184] Shukri R., Mohamed S., Mustapha N.M. (2010). Cloves Protect the Heart, Liver and Lens of Diabetic Rats. Food Chem..

[185] de Souza K.A., de Oliveira Monteschio J., Mottin C., Ramos T.R., de Moraes Pinto L.A., Eiras C.E., Guerrero A., do Prado I.N. (2019). Effects of Diet Supplementation with Clove and Rosemary Essential Oils and Protected Oils (Eugenol, Thymol and Vanillin) on Animal Performance, Carcass Characteristics, Digestibility, and Ingestive Behavior Activities for Nellore Heifers Finished in Feedlot. Livest. Sci..

[186] Arcaro C.A., Gutierres V.O., Assis R.P., Moreira T.F., Costa P.I., Baviera A.M., Brunetti I.L. (2014). Piperine, a Natural Bioenhancer, Nullifies the Antidiabetic and Antioxidant Activities of Curcumin in Streptozotocin-Diabetic Rats. PLoS ONE.

[187] Stobiecka M., Król J., Brodziak A., Klebaniuk R., Kowalczuk-Vasilev E. (2023). Effects of Supplementation with an Herbal Mixture on the Antioxidant Capacity of Milk. Animals.

[188] Boonrueng P., Wasana P.W.D., Hasriadi, Vajragupta O., Rojsitthisak P., Towiwat P. (2022). Combination of Curcumin and Piperine Synergistically Improves Pain-like Behaviors in Mouse Models of Pain with No Potential CNS Side Effects. Chin. Med..

[189] Zanzer Y.C., Batista Â.G., Dougkas A., Tovar J., Granfeldt Y., Östman E. (2019). Difficulties in Translating Appetite Sensations Effect of Turmeric-Based Beverage When given Prior to Isoenergetic Medium- or High-Fat Meals in Healthy Subjects. Nutrients.

[190] Gregersen N.T., Belza A., Jensen M.G., Ritz C., Bitz C., Hels O., Frandsen E., Mela D.J., Astrup A. (2013). Acute Effects of Mustard, Horseradish, Black Pepper and Ginger on Energy Expenditure, Appetite, Ad Libitum Energy Intake and Energy Balance in Human Subjects. Br. J. Nutr..

[191] Zanzer Y.C., Plaza M., Dougkas A., Turner C., Björck I., Östman E. (2017). Polyphenol-Rich Spice-Based Beverages Modulated Postprandial Early Glycaemia, Appetite and PYY after Breakfast Challenge in Healthy Subjects: A Randomized, Single Blind, Crossover Study. J. Funct. Foods.

[192] Khan S., Arif M., Laraib H., Naqvi S.N., Shah O.A., Farooq U., Sami-Ullah M., Khan G.A. (2024). The Effect of Turmeric and Black Pepper Powder Incorporated in Breakfast on Postprandial Glycemia, Appetite, Palatability, and Gastrointestinal Well-Being in Normal-Weight Adults. Food Sci. Nutr..

[193] Gostiljac D.M., Popovic S.S., Dimitrijevic-Sreckovic V., Ilic S.M., Jevtovic J.A., Nikolic D.M., Soldatovic I.A. (2024). Effect of Special Types of Bread with Select Herbal Components on Postprandial Glucose Levels in Diabetic Patients. World J. Diabetes.

[194] Nakayama H., Tsuge N., Sawada H., Masamura N., Yamada S., Satomi S., Higashi Y. (2014). A Single Consumption of Curry Improved Postprandial Endothelial Function in Healthy Male Subjects: A Randomized, Controlled Crossover Trial. Nutr. J..

[195] Li Z., Henning S.M., Zhang Y., Zerlin A., Li L., Gao K., Lee R.P., Karp H., Thames G., Bowerman S. (2010). Antioxidant-Rich Spice Added to Hamburger Meat during Cooking Results in Reduced Meat, Plasma, and Urine Malondialdehyde Concentrations. Am. J. Clin. Nutr..

[196] Li Z., Henning S.M., Zhang Y., Rahnama N., Zerlin A., Thames G., Tseng C.H., Heber D. (2013). Decrease of Postprandial Endothelial Dysfunction by Spice Mix Added to High-Fat Hamburger Meat in Men with Type 2 Diabetes Mellitus. Diabet. Med..

[197] Haldar S., Chia S.C., Henry C.J. (2018). Polyphenol-Rich Curry Made with Mixed Spices and Vegetables Increases Postprandial Plasma GLP-1 Concentration in a Dosedependent Manner. Eur. J. Clin. Nutr..

[198] Haldar S., Lim J., Chia S.C., Ponnalagu S., Henry C.J. (2018). Effects of Two Doses of Curry Prepared with Mixed Spices on Postprandial Ghrelin and Subjective Appetite Responses—A Randomized Controlled Crossover Trial. Foods.

[199] Haldar S., Chia S.C., Lee S.H., Lim J., Leow M.K.S., Chan E.C.Y., Henry C.J. (2019). Polyphenol-Rich Curry Made with Mixed Spices and Vegetables Benefits Glucose Homeostasis in Chinese Males (Polyspice Study): A Dose–Response Randomized Controlled Crossover Trial. Eur. J. Nutr..

[200] Khine W.W.T., Haldar S., De Loi S., Lee Y.K. (2021). A Single Serving of Mixed Spices Alters Gut Microflora Composition: A Dose–Response Randomised Trial. Sci. Rep..

[201] Skulas-Ray A.C., Kris-Etherton P.M., Teeter D.L., Chen C.Y.O., Heuvel J.P.V., West S.G. (2011). A High Antioxidant Spice Blend Attenuates Postprandial Insulin and Triglyceride Responses and Increases Some Plasma Measures of Antioxidant Activity in Healthy, Overweight Men. J. Nutr..

[202] McCrea C.E., West S.G., Kris-Etherton P.M., Lambert J.D., Gaugler T.L., Teeter D.L., Sauder K.A., Gu Y., Glisan S.L., Skulas-Ray A.C. (2015). Effects of Culinary Spices and Psychological Stress on Postprandial Lipemia and Lipase Activity: Results of a Randomized Crossover Study and in Vitro Experiments. J. Transl. Med..

[203] Oh E.S., Petersen K.S., Kris-Etherton P.M., Rogers C.J. (2020). Spices in a High-Saturated-Fat, High-Carbohydrate Meal Reduce Postprandial Proinflammatory Cytokine Secretion in Men with Overweight or Obesity: A 3-Period, Crossover, Randomized Controlled Trial. J. Nutr..

[204] Petersen K.S., Rogers C.J., West S.G., Proctor D.N., Kris-Etherton P.M. (2020). The Effect of Culinary Doses of Spices in a High-Saturated Fat, High-Carbohydrate Meal on Postprandial Lipemia and Endothelial Function: A Randomized, Controlled, Crossover Pilot Trial. Food Funct..

